# The transcriptomic signature of different sexes in two protogynous hermaphrodites: Insights into the molecular network underlying sex phenotype in fish

**DOI:** 10.1038/s41598-018-21992-9

**Published:** 2018-02-23

**Authors:** A. Tsakogiannis, T. Manousaki, J. Lagnel, A. Sterioti, M. Pavlidis, N. Papandroulakis, C. C. Mylonas, C. S. Tsigenopoulos

**Affiliations:** 1 Institute of Marine Biology, Biotechnology and Aquaculture (IMBBC), Hellenic Centre for Marine Research (H.C.M.R.), Heraklion, Greece; 2 Department of Biology, University of Crete, Heraklion, Greece

## Abstract

Sex differentiation is a puzzling problem in fish due to the variety of reproductive systems and the flexibility of their sex determination mechanisms. The Sparidae, a teleost family, reflects this remarkable diversity of sexual mechanisms found in fish. Our aim was to capture the transcriptomic signature of different sexes in two protogynous hermaphrodite sparids, the common pandora *Pagellus erythrinus* and the red porgy *Pagrus pagrus* in order to shed light on the molecular network contributing to either the female or the male phenotype in these organisms. Through RNA sequencing, we investigated sex-specific differences in gene expression in both species’ brains and gonads. The analysis revealed common male and female specific genes/pathways between these protogynous fish. Whereas limited sex differences found in the brain indicate a sexually plastic tissue, in contrast, the great amount of sex-biased genes observed in gonads reflects the functional divergence of the transformed tissue to either its male or female character. Α common “crew” of well-known molecular players is acting to preserve either sex identity of the gonad in these fish. Lastly, this study lays the ground for a deeper understanding of the complex process of sex differentiation in two species with an evolutionary significant reproductive system.

## Introduction

The differences between sexes have always fascinated, yet troubled philosophers and scientists^1^. Unlike the commonality of sexual reproduction, the ways that species express their sexuality vary considerably in the animal kingdom. Among the animals that reproduce sexually, teleost fishes show a very wide repertoire of reproductive modes, including all known reproductive styles found in vertebrates, from gonochorism (*i.e*., after sex is determined, it remains unchanged for the entire life cycle) to unisexuality (all-female species) and hermaphroditism (sequential, serial and simultaneous, including outcrossing and selfing species)^2,3^. These diverse sexual phenotypes in fish are regulated by a variety of sex determination (SD) mechanisms, along a continuum of environmental and genetic factors^3,4^.

The knowledge concerning sex differences at the molecular level results mainly from studies in humans and model vertebrates such as mouse, chicken and African clawed frog^5–8^. In fish, only few genes have been reported so far to play the role of master regulators in the SD systems of an investigated species. These include *DmY/Dmrt1bY* in *Oryzias latipes* and in *Oryzias curvinotus*^9^, *Gsdf i*n *Oryzias luzonensis*^10^, *Amhy* in *Odontesthes hatcheri*^11^, *Amhr2* in *Takifugu rubripes*, *T. pardalis* and *T. poecilonotus*^12^ and *SdY* in the salmonid family^13^. Recently, a distant cis-regulatory element of *Sox3* necessary for male determination in *Oryzias dancena* (XX/XY SD system) has also been identified^14^ and *Dmrt1* has been suggested as the sex-determining gene (SDG) in *Cynoglossus semilaevis* (ZZ/ZW SD system)^15^. To date, studies regarding the sex-specific differences in gene expression have been conducted mainly in SD systems of model fish species that are well characterized at the genomic level, with distinguishable heteromorphic sex chromosomes, exhibiting Genetic Sex Determination (GSD) and gonochorism^16–18^. The flexibility governing reproductive mechanisms in fish, with the presence of different systems among even phylogenetically close species makes the picture more complicated. However, sex-linked markers and/or sex-determining regions have already been reported in several species, including some that are of interest to the aquaculture industry, such as the catfish *Clarias gariepinus*^19^, the Nile tilapia *Oreochromis niloticus*^20^, the rainbow trout *Oncorhynchus mykiss*^21^, the turbot *Scophthalmus maximus*^22^, the half-smooth tongue sole *Cynoglossus semilaevis*^23^, some salmonids^24–26^, Gasterosteidae^27^, Eigenmannia^28^ and the European sea bass *Dicentrarchus labrax*^29^.

Among teleosts, the Sparidae family is considered to be one of the most diversified families regarding reproductive systems, exhibiting nearly all sexual modes known in fish^30^. In addition to gonochoristic species, various forms of hermaphroditism have been recorded^31^. Therefore, the Sparidae family is a unique model for comparative studies to understand the molecular mechanisms underlying different sexual motifs. Until recently, genetic studies on sex determination/differentiation and sex change in sparids were conducted mainly on the protandrous black porgy *Acanthopagrus schlegelii*^32,33^ and some QTL studies have been carried out in gilthead seabream *Sparus aurata*^34^. The first work on sex-specific gene expression in a sparid was on a rudimentary hermaphrodite sharpsnout seabream *Diplodus puntazzo*^35^. Despite the fact that hermaphroditism is widespread in many fish lineages^36^, studies on sex-related transcriptomic differences have been conducted in only a few hermaphrodites: the protandrous Asian seabass *Lates calcarifer*^37^, the protogynous bluehead wrasse *Thalassoma bifasciatum*^38^ and, very recently the protogynous clownfish *Amphiprion bicinctus*^39^.

The common pandora *Pagellus erythrinus* and the red porgy *Pagrus pagrus* are two other sparids exhibiting protogynous hermaphroditism. They are both benthopelagic fish, distributed through the Eastern Atlantic Ocean along the west coast of Europe and Africa (from Scandinavia to Angola), the entire Mediterranean Sea and the Black Sea. The red porgy’s distribution extends also to the western Atlantic, from the northern USA to Argentina. Typically, both fishes mature first as females, changing to males after two years of age or when attaining a body length of 17–18 cm for common pandora and after three years of age or about 24 cm of body length for red porgy^40,41^. However, sexual patterns appear to be far more complex. Sex change and the sexual structure of populations of sequential hermaphrodite fishes may be genetically controlled or triggered by environmental/external factors, such as behavioral or/and demographic changes in their social systems^42^, or even a combination of both^43^. According to Cataudella *et al*.^44^, most of the Mediterranean sparids have a haploid chromosome number of 24 and recently a linkage map, consisting of 24 LGs, was built for the common pandora^45^. To our knowledge, there is no evidence concerning the presence of heteromorphic sex chromosomes in these sparid species or whether their sex determination system is genetic, environmental or an amalgam of both. Because of the intriguing reproductive mode of common pandora and the red porgy, they are important to be studied from an evolutionary point of view, as protogyny has been reported as the most common model of hermaphroditism in teleosts^46–48^. Moreover, their potential for large-scale aquaculture covers the need for species diversification in the sector^40^.

This study presents the transcriptomic signature of different sexes in these two sparid fishes, which share the same protogynous reproductive mode. Using RNA sequencing^49^, we analyzed and compared differences between male and female transcriptomes of common pandora and red porgy. In particular, we investigated gene expression differences between male and female gonads and brains, the two most important tissues in sexual development and reproduction^50^. We obtained a global view of sex-biased expression in these tissues and through comparative analysis we also revealed common male and female specific genes/pathways that are implicated in sex differentiation and sex maintenance. We also carefully investigated the presence of genes reported to be involved in sex determination/differentiation mechanisms in other vertebrates and fish and compared their expression patterns in the two species under study. Our results contribute to the understanding of the complex processes of sex differentiation in these protogynous hermaphrodites.

## Results

### Pre-processing treatment of reads and assembly construction

Illumina HiSeq. 2500 sequencing of red porgy and common pandora samples yielded 685,707,304 (342,853,652 read pairs) and 644,189,592 (322,094,796 read pairs) reads, respectively. The raw sequence data have been submitted to the National Centre for Biotechnology Information (NCBI) Sequence Read Archive (SRA) database (BioProject ID: PRJNA395994).

For red porgy, pre-processing of reads resulted in 247,712,854 high-quality reads used for the initial transcriptome assembly, which consisted of 295,367 putative transcripts (N50: 1,961 nucleotides, mean length: 928 nucleotides) and showed significant similarity to 23,122 out of 26,754 European sea bass genes. After transcript quality assessment, our initial dataset was limited to 98,012 contigs (N50: 2,978; mean length: 1,587 nucleotides) corresponding to 67,132 genes and was significantly similar to 20,130 European sea bass genes. Filtering resulted in elimination of 197,355 possibly spurious transcripts and this restricted dataset constituted the final transcriptome with only slightly reduced numbers of hits to the unique genes of European sea bass.

**Figure 1:**
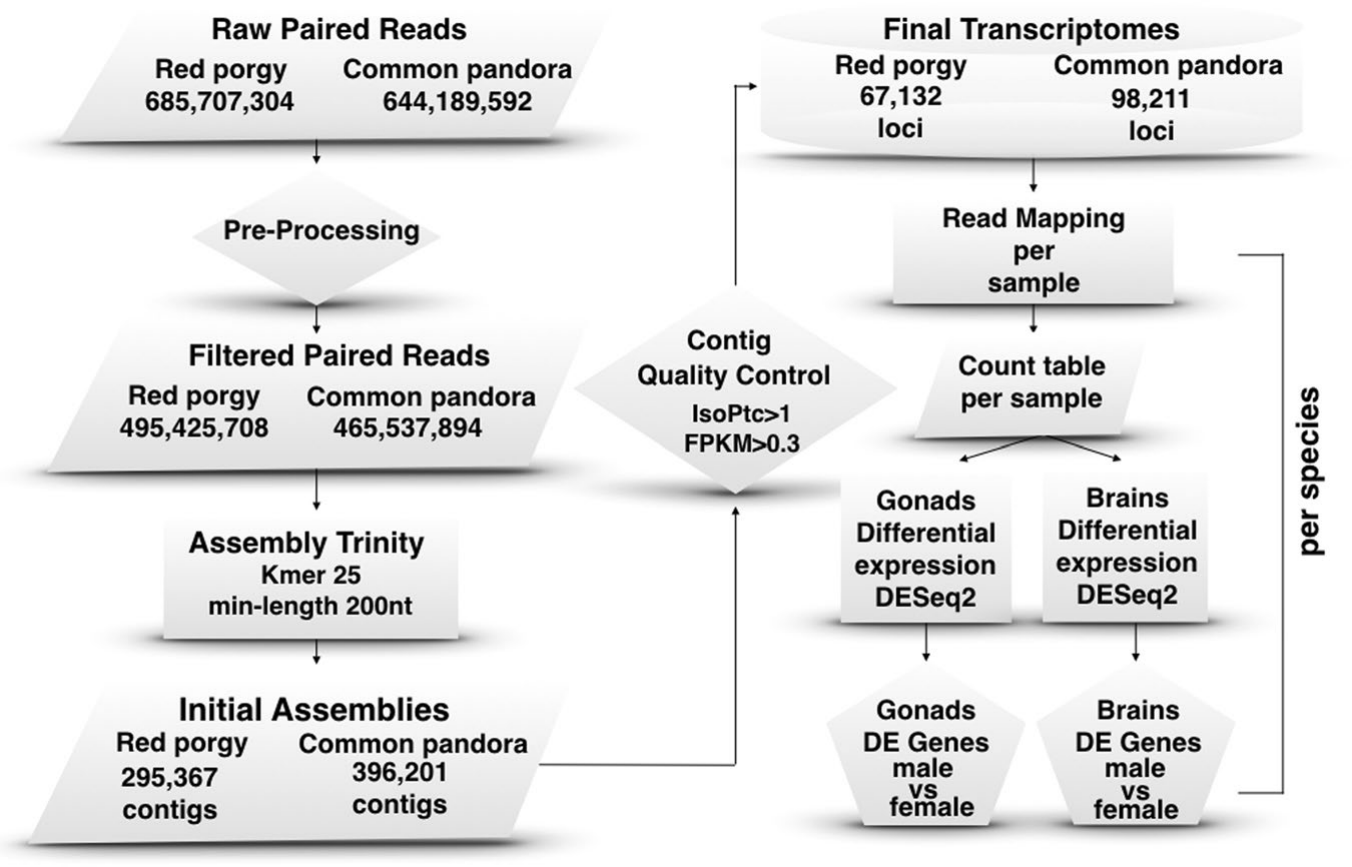
The pipeline followed to build assemblies and differential expression profiles in brains and gonads of two protogynous sparids. Flow chart of the basic steps implemented from raw reads for the selection of final assembled loci and differentially expressed genes in brains and gonads of both species.

For common pandora, filtering of reads resulted in 232,768,947 high-quality reads that were used to construct an initial assembly of 396,201 putative transcripts (N50: 1,529 nucleotides, mean length: 824 nucleotides). Additional editing excluded 254,892 possibly spurious transcripts, thus limiting our dataset to 141,309 contigs (N50: 2,450; mean length: 1,254 nucleotides), corresponding to 98,211 genes. On this occasion, the unfiltered transcript dataset showed significant similarity to 23,631 European sea bass genes out of 26,754, while the filtered assembly showed significant similarity to 21,028 genes. Following the same reasoning, the restricted dataset constituted the final transcriptome (Fig. 1).

To evaluate the accuracy of the assembled sequences (transcripts), all the usable sequencing reads were aligned onto the transcripts using Bowtie2^51^. A high percentage of red porgy and common pandora reads (84% and 81%, respectively) were successfully back-mapped on each assembly. Similarities between assemblies and UniProtKB/SwissProt database disclosed 21,217 and 21,251 contigs for red porgy and common pandora, respectively, with coverage higher than 80%. In addition, BUSCO run (Supplementary Table ST1) indicated that the assemblies were nearly complete with 86% and 82% complete matches of vertebrate orthologs, found in red porgy and common pandora assemblies, respectively.

### Transcriptome annotation

**Figure 2:**
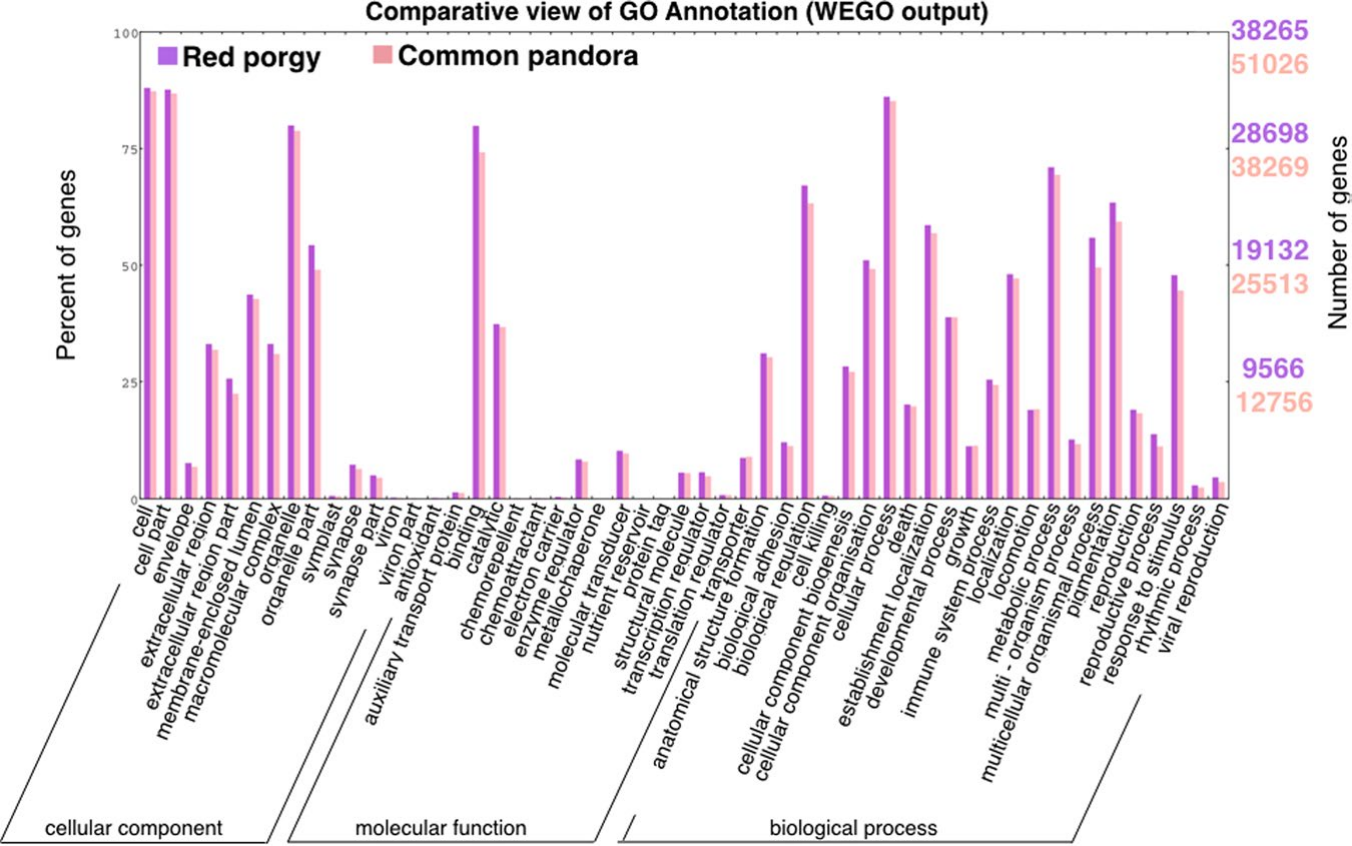
Comparative view of GO annotation. Comparison of Gene Ontology. (GO) categories (assigned by WEGO) between red porgy and common pandora. GO terms for the genes of red porgy (dark purple) and common pandora (pink) were categorized into cellular components, molecular functions and biological processes.

The blastx search in red porgy and common pandora assembled transcriptomes (*e* -value cut-off <10^−5^) returned 43,123 and 50,106 contigs, with at least one blast hit against Uniprot^52^ and 38,354 and 44,392 contigs with a protein domain match against InterProScan^53^ search, respectively (Supplementary Table ST2). To identify the possible functions, Gene Ontology (GO) assignments were used to classify the sequences. Total numbers of 38,265 red porgy contigs and 51,026 common pandora contigs with at least one GO term were found (Blast2Go^54^ annotated) (Supplementary Table ST2). The sequences from both species were categorized to thirteen molecular function (MF), 22 biological process (BP) and thirteen cellular component (CC) gene categories in GO level 2 (general function categories) (Supplementary Figures SF1–4). To assess the functional diversity of the assembled transcriptome, GO annotations of red porgy were compared with those of common pandora transcriptome, reflecting a similar functional distribution on GO categories and similar sequence diversity of the two transcriptomes. Profiling of both species (whole transcriptome) was generally very much alike, as shown in the comparative view produced by WEGO^55^ (Fig. 2).

The potential enzymes were characterized based on the chemical reaction they catalyze, using the prediction of Enzyme Commission (EC) numbers for each sequence. For red porgy, among the 38,265 blast2go-annotated contigs, 9,572 contigs were classified to 1,194 different EC numbers, while among the 51,026 common pandora blast2go-annotated contigs, 12,821 were classified to 1,243 different EC numbers (Supplementary Table ST2).

### Global expression pattern of brains and gonads

**Figure 3:**
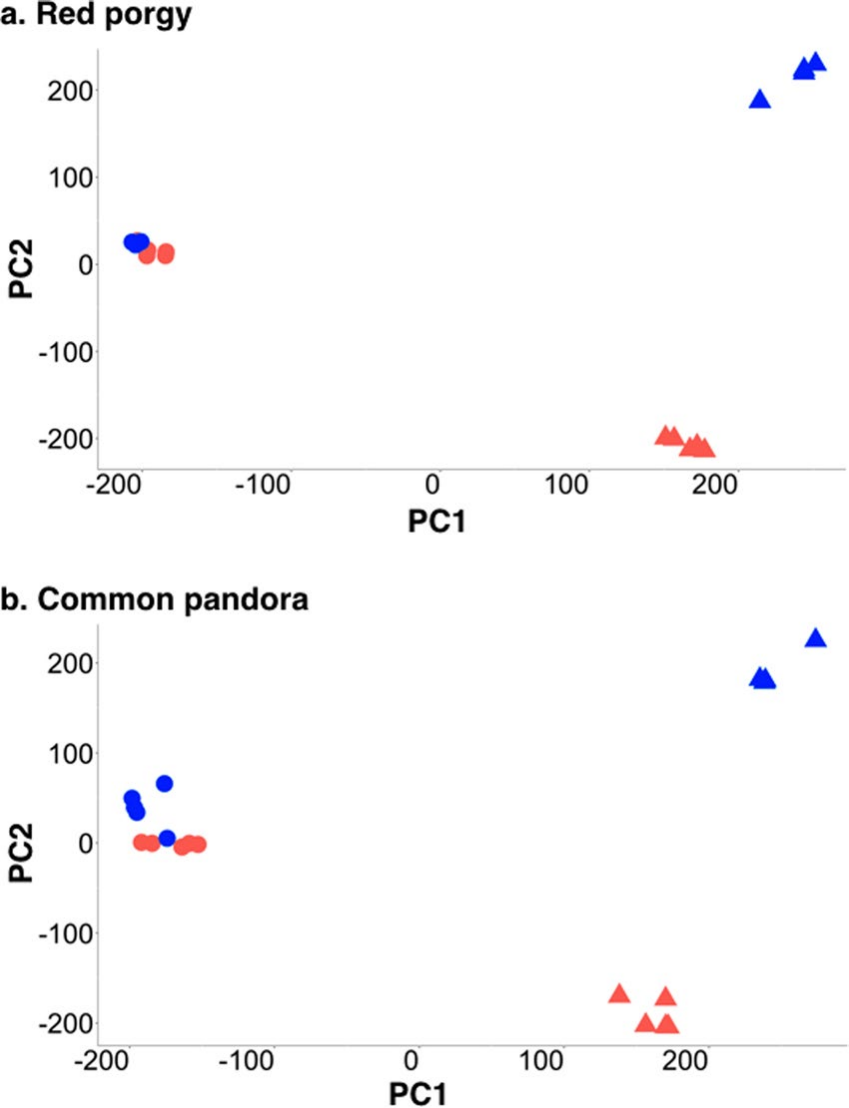
Principal component analysis of global expression profiles in two protogynous sparids. Principal component analysis of overall expression profiles of the gonad and brain samples of male and female individuals (**a**) Red porgy (PC1 = 49% variance and PC2 = 27% variance), (**b**) Common pandora (PC1 = 44% variance and PC2 = 22% variance). Blue = males, Red = females and Triangles = Gonads, Circles = Brains). All genes with non-zero total read count in the count matrix used for the PCA plot (a filtered matrix).

The principal component analysis (PCA) plots obtained for the global gene expression pattern observed in brain and gonad tissues of the nine red porgy and ten common pandora individuals showed the formation of three separate clusters for both species: the brains, the male gonads (testes) and the female gonads (ovaries) (Fig. 3). While testes and ovaries showed a clear separation with a remarkable variation within the two groups, male or female brains from both species were clustered together with very similar expression patterns.

Tissue-specific gene ontology profiling for both fish revealed differences in GO terms representation between brains and gonads, in all three categories in GO level 2. When comparing brains and gonads, except from altered percentages of the same main ontology categories (shared between brains and gonads), there were terms such as *synapse (CC), cell junction (CC), behavior (BP), rhythmic process (BP), cell aggregation (BP), cell killing (BP), chemoattractant activity (MF)* and *chemorepellent activity (MF)* that were present only in the brains of the two sparids, but were totally absent or present in low numbers in their gonads. This would indicate brain specificity of the underlying genes (Supplementary Figures SF1–4).

**Figure 4:**
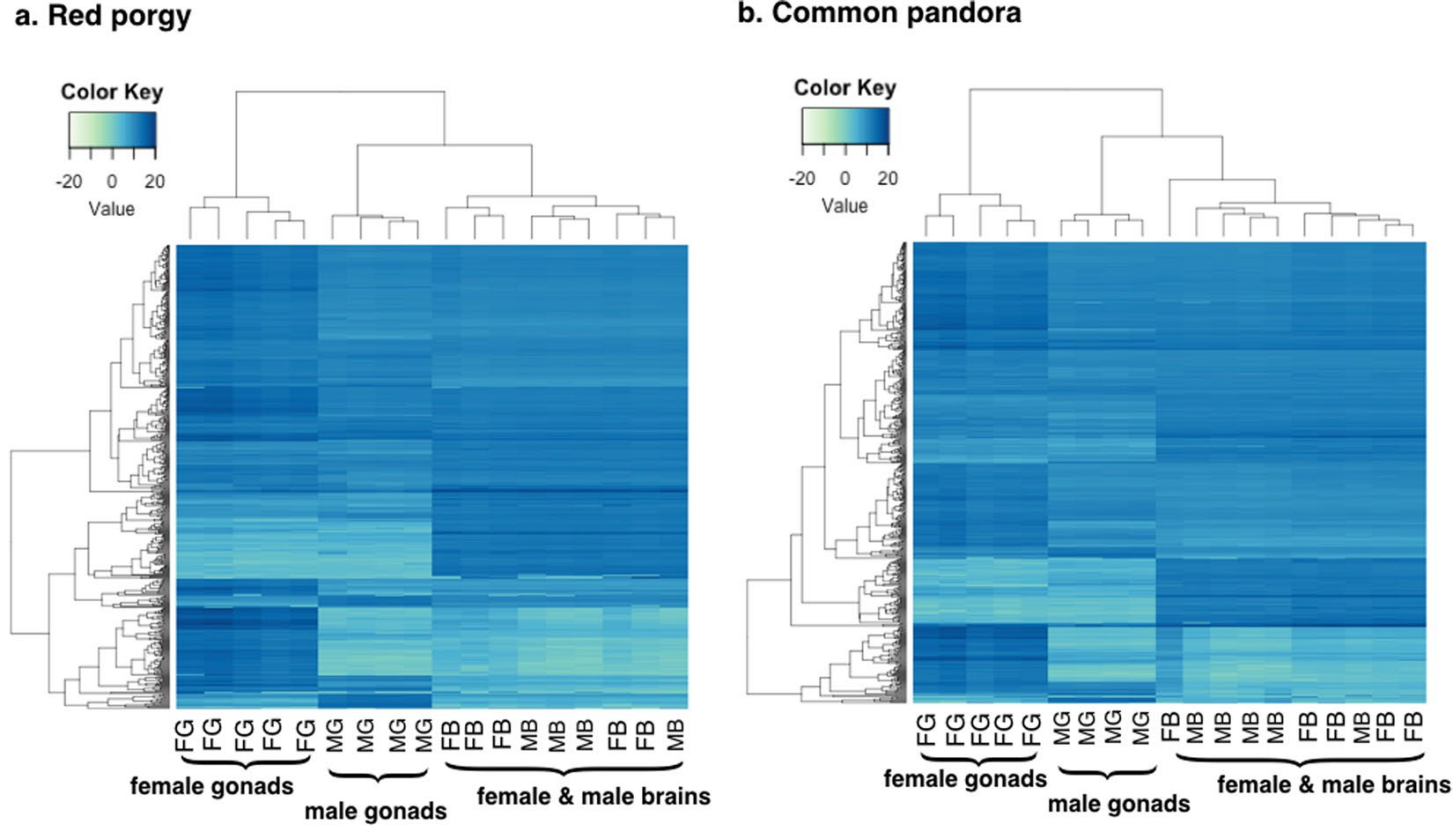
Heatmap plots for global expression profiles in two protogynous sparids. Heatmaps of the variance stabilized, transformed count data for male and female brains and gonads. (**a**) Red porgy, (**b**) Common pandora samples clustered according to their global gene expression patterns. Only the top 500 variable genes were used to produce the heatmaps for visibility reasons.

For a better understanding of the biological processes and molecular functions underlying the nature of a male or female phenotype, GO term enrichment analysis was carried out in order to detect differentially-expressed (DE) genes over-represented in the different sexes of both species. Here, the term ‘enrichment’ stands for the comparison between the DE genes of the female (test-set) against the male (reference-set) either for brains or gonads. The same picture of sample clustering in both species was also shown through heatmap plots (Fig. 4). Comparisons between males and females were conducted separately for the brain and gonad samples to produce PCA plots and heatmaps (Supplementary Figures SF5 and SF6). This closer view again showed that sex-dependent expression differences are greater by far in gonads than in brains. In the brain PCA plots of both species, males and females clustered together, with red porgy brain samples clustered more tightly than those of the common pandora that show a looser clustering.

### Comparing gene expression in male and female brains

**Figure 5:**
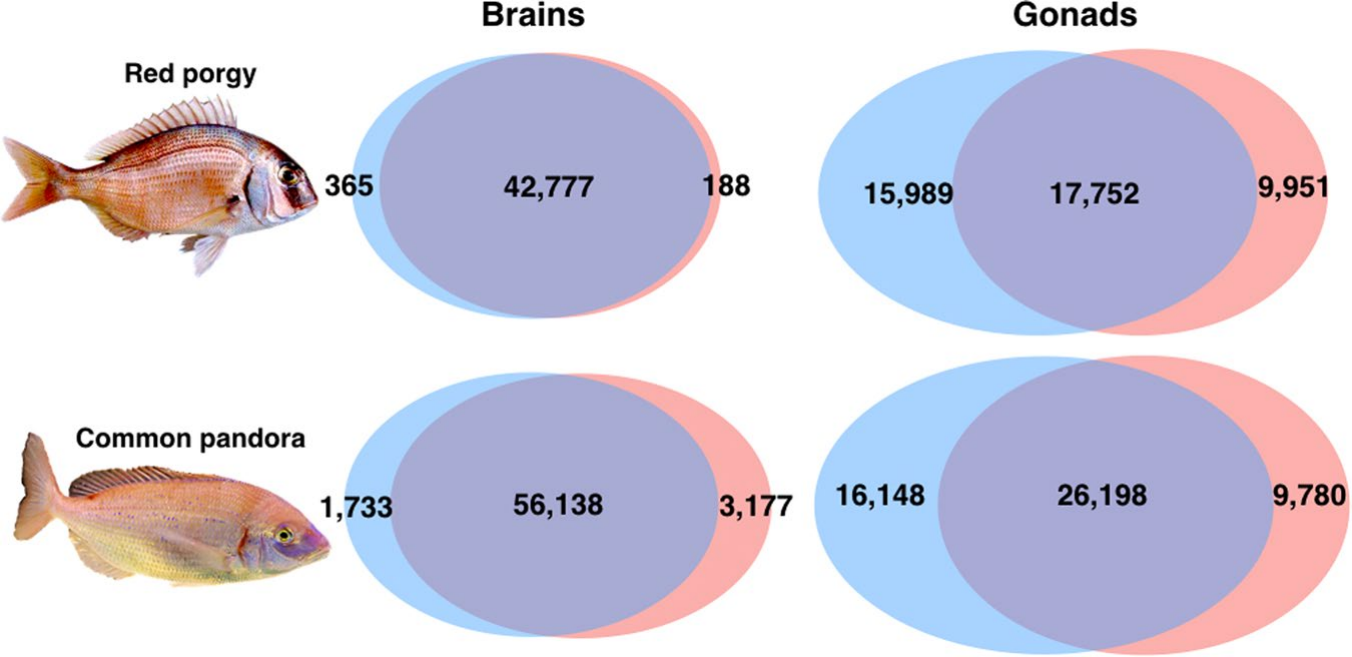
Comparative view of the number of genes differently expressed in brains and gonads of males and females of two protogynous sparids. Venn-like diagram showing the genes differentially expressed in brains and gonads of male and female red porgy and common pandora. Light-Blue: male-biased genes. Red: female-biased genes. Dark-Purple (intersection): un-biased genes.

Out of 67,132 red porgy and 98,211 common pandora total loci, 43,330 and 61,048 genes had at least 15 reads in all compared samples (the sum of counts in each row of the count matrix >15) and were used for differential expression analysis. In general, brain DE genes showed low counts and low fold-change differences (Supplementary Figure SF7). For red porgy brain samples, 553 genes differed significantly with 365 of them having a male-biased (over-expression) and 188 of them having female-biased expression. Common pandora brain samples showed higher numbers of DE genes and an opposite direction of expression than red porgy brain samples. In total, 4,910 genes showed differential expression between the sexes, of which 1,733 were up-regulated in the male brains and 3,177 of them were up-regulated in the female brains (Fig. 5).

A GO analysis was conducted for all three general function categories (BP, MF, CC). Focusing on biological processes, brain enrichment analysis resulted in 241 GOs over-represented in female (light red-colored in Supplementary Excel Table SET1) and 265 in male common pandora (light blue-colored in Supplementary Excel Table SET1). For red porgy, 58 GOs were over-represented in females (light red-colored in Supplementary Excel Table SET2) and 113 in males (light blue-colored in Supplementary Excel Table SET2).

### Comparing gene expression in male and female gonads

In gonad samples, we applied the same filter and kept only loci with a sum of expected counts in each row of the count matrix >15. Specifically, 43,692 and 52,126 genes fulfilled this criterion and were examined for differential expression in red porgy and common pandora gonads, respectively. The analysis revealed striking differentiation between testes and ovaries. In total, 18,724 red porgy gonadal genes showed a significant differential expression, of which 15,989 were up-regulated in the testes and 9,951 of them were up-regulated in the ovaries. In the case of common pandora, 25,928 gonadal genes had significantly differential expression, 16,148 them with up-regulation in their testes and 9,780 them with up-regulation in their ovaries (Fig. 5).

Focusing again on biological processes, Enrichment Analysis of DE genes in common pandora gonad tissues resulted in 236 GO terms significantly enriched in testes (light blue-colored in Supplementary Table SET3) while 517 GO terms were found in ovaries (light red-colored in Supplementary Table SET3); likewise, in red porgy 284 GO terms were significantly enriched in testes (light blue-colored in Supplementary Table SET4) and ovaries enriched with 370 GO terms (light red-colored in Supplementary Table SET4).

### Sex-specific candidate genes in gonads

To have a closer view in the molecular playground causing sex dimorphism in these sparids, we checked the presence and the expression patterns in their gonads, of genes that have been reported as having an important role or participating in sex determination/differentiation or in sex maintenance in other fish or vertebrates. We selected 114 candidate genes in total. Out of this list, 84 genes were present in the transcriptomes of both species, while *Srd5a2* was present only in red porgy transcriptome and *Dmrt3a* was found only in common pandora. Table 1 includes all the genes with significantly differential expression in at least one of the studied species (excluding the not significantly different -NSD- characterized- genes in both species) and compares their expression pattern between them.

**Table 1:** Expression pattern of gonadal genes implicated in sex determination/differentiation of common pandora and red porgy.

Gene name	Gene description	Gene ID		Expression			
		Common pandora	Red porgy	Common pandora		Red porgy	
				sex	log2FC	sex	log2FC
Cyp19a1	Aromatase a (gonad isoform)	TR72019|c0_g1	TR50491|c0_g1	**F**	−8.12	**F**	−5.33
Gdf9	Growth and differentiation factor 9	TR70663|c0_g1	TR52139|c0_g1	**F**	−7.36	**F**	−8.35
Sox3	SRY-related HMG box 3	TR69218|c2_g2	TR64334|c1_g1	**F**	−6.91	**F**	−7.45
LHb	Luteinizing hormone, beta polypeptide	TR66625|c0_g1	TR40794|c1_g1	**F**	−6.54	**F**	−9.67
Foxl2	Forkhead box L2	TR59502|c0_g1	TR43506|c0_g1	**F**	−6.18	**F**	−7.59
Figla	Factor in the germline alpha	TR54099|c0_g1	TR49469|c0_g1	**F**	−5.77	**F**	−9.09
Wnt4a	Wingless-type MMTV integration site family, member 4a	TR67106|c0_g2	TR40636|c0_g1	**F**	−5.61	**F**	−7.02
Cyp26a1	Cytochrome P450, family 26, subfamily a, polypeptide 1	TR72448|c0_g1	TR49995|c0_g1	**F**	−4.60	**F**	−6.73
Dmrt2/Dmrt2a	Doublesex- and mab-3-related transcription factor 2(a)	TR78139|c1_g1	TR59371|c2_g1	**F**	−4.52	**F**	−7.92
Lhr	Luteinizing hormone receptor	TR89660|c1_g2	TR61712|c0_g1	**F**	−4.40	**F**	−3.84
Stra6	Stimulated by retinoic acid gene 6	TR86270|c0_g1	TR59700|c3_g1	**F**	−3.99	**F**	−5.45
Arb	Androgen receptor beta	TR85026|c0_g1	TR60273|c1_g1	**F**	−3.77	**F**	−4.16
Sox10	SRY-related HMG box 10	TR79411|c5_g2	TR64695|c1_g1	**F**	−3.48	**NSD**	(−)
WT1b	Wilms tumor protein 1b	TR89074|c1_g1	TR57899|c0_g1	**F**	−3.40	**F**	−2.96
Dax1	Dosage-sensitive sex reversal, adrenal hypoplasia critical region, on chromosome X, gene 1	TR19608|c0_g1	TR64306|c1_g1	**F**	−2.93	**F**	−0.74
Gata-4	Gata-binding protein 4	TR74516|c2_g1	TR63369|c1_g1	**F**	−2.92	**NSD**	(−)
Sf1/FTZ-F11	Steroidogenic factor-1/fushi tarazu factor-1	TR80163|c1_g2	TR56092|c1_g1	**F**	−2.88	**F**	−2.29
Star-like	Steroidogenic acute regulatory protein	TR52301|c0_g1	TR53314|c0_g1	**F**	−2.80	**NSD**	(−)
WT1	Wilms tumor protein 1a	TR89074|c0_g1	TR67279|c4_g5	**F**	−2.79	**F**	−2.41
Pdgfba	Platelet-derived growth factor beta a	TR86429|c1_g1	TR58625|c0_g1	**F**	−2.62	**F**	−1.97
Fst/Fsta	Follistatin	TR66770|c0_g3	TR48089|c0_g1	**F**	−2.37	**F**	−2.67
Pdgfab	Platelet-derived growth factor alpha b	TR75590|c0_g1	TR64531|c0_g1	**F**	−2.20	**F**	−1.74
Pdgfrb2	Platelet-derived growth factor receptor, beta 2	TR83411|c3_g1	TR55622|c2_g1	**F**	−2.18	**F**	−3.33
Hsd17b1	17β-Hydroxysteroid dehydrogenase type 1	TR82119|c0_g3	TR59823|c0_g1	**F**	−2.11	**F**	−6.02
Hdac2	Histone deacetylase 2	TR66800|c1_g1	TR56322|c1_g2	**F**	−2.11	**F**	−3.04
Dhh	Desert hedgehog	TR84448|c0_g1	TR59771|c0_g1	**F**	−2.09	**NSD**	(−)
Esr2b/Esrrb2	Estrogen receptor 2b	TR77491|c0_g1	TR52662|c0_g1	**F**	−1.97	**NSD**	(−)
Esrrb	Estrogen related receptor beta	TR89871|c1_g3	TR66216|c1_g1	**F**	−1.91	**F**	−4.07
Ara/Ar	Androgen receptor/Androgen receptor alpha	TR83707|c1_g2	TR60985|c0_g1	**F**	−1.89	**F**	−2.70
Hdac7	Histone deacetylase 7	TR84823|c3_g1	TR57004|c0_g1	**F**	−1.77	**F**	−1.16
Srd5a3	5α-reductase 3	TR83935|c0_g1	TR68483|c0_g2	**F**	−1.53	**NSD**	(−)
Hdac10	Histone deacetylase 10	TR88916|c0_g6	TR67490|c0_g1	**F**	−1.46	**F**	−1.42
Dnmt3abc1	DNA methyltransferase 3ab_contig 1	TR78244|c1_g1	TR53015|c0_g1	**F**	−1.29	**F**	−1.42
Ctnnb1	Catenin (cadherin-associated protein), beta 1	TR64263|c0_g2	TR46279|c0_g1	**F**	−0.80	**F**	−1.22
Pdgfra	Platelet-derived growth factor receptor, alpha	TR88623|c1_g1	TR66258|c1_g1	**F**	−0.76	**NSD**	(−)
Dnmt1	DNA methyltransferase 1	TR83768|c0_g1	TR62712|c0_g1	**F**	−0.72	**F**	−0.85
DDX11	DEAD/H box helicase 11	TR90413|c2_g1	TR66969|c0_g1	**M**	0.86	**M**	0.80
Raraa	Retinoid acid receptor alpha a	TR82123|c4_g2	TR36645|c1_g1	**M**	0.95	**M**	1.61
Tdrd1	Tudor domain containing 1	TR91962|c2_g5	TR68477|c1_g4	**M**	0.96	**NSD**	(−)
Nr3c1	Glucocorticoid receptor	TR84453|c3_g1	TR63170|c1_g1	**M**	1.13	**M**	1.60
Tdrd7	Tudor domain containing 7	TR88488|c1_g2	TR63911|c0_g1	**M**	1.25	**M**	1.81
Esrra1	Estrogen-related receptor alpha	TR86271|c1_g1	TR60672|c1_g2	**M**	1.55	**M**	1.55
Srd5a1	Steroid-5-alpha-reductase, alpha polypeptide 1	TR91999|c2_g1	TR64324|c0_g1	**M**	1.58	**NSD**	(−)
Sox9	SRY-related HMG box 9	TR69398|c2_g1	TR59011|c5_g2	**M**	1.91	**M**	1.25
Dnmt3aa	DNA methyltransferase 3aa	TR73384|c7_g1	TR64267|c0_g1	**M**	2.09	**M**	4.11
Ep300a	Histone acetyltransferase—E1A binding protein 300a	TR84051|c2_g6	TR60830|c1_g1	**M**	2.77	**M**	2.49
Amh	Anti-Müllerian hormone or Müllerian-inhibiting substance	TR91164|c5_g4	TR66874|c1_g1	**M**	2.89	**NSD**	(−)
Hdac8	Histone deacetylase 8	TR86781|c2_g5	TR62362|c1_g1	**M**	3.04	**M**	2.73
Fstl5	Follistatin-like 5	TR77519|c0_g5	TR60004|c0_g1	**M**	3.22	**M**	3.15
Rarb	Retinoid acid receptor beta	TR81734|c1_g3	TR65946|c1_g2	**M**	3.58	**M**	2.99
Dmrt3/Dmrt3a	Double sex and mab-3 related transcription factor 3(a)	TR67762|c1_g1	(−)	**M**	3.80	**(−)**	
Cyp11c1/Cyp11b	Cytochrome P450, family 11, subfamily C, polypeptide 1/11beta-hydroxylase	TR72664|c0_g1	TR47689|c0_g1	**M**	5.34	**NSD**	(−)
Dmrt1	Doublesex- and mab-3-related transcription factor 1	TR70670|c0_g1	TR67635|c0_g1	**M**	6.74	**M**	6.21
Hsd11b3	11β-Hydroxysteroid dehydrogenase type 3	TR71766|c4_g1	TR60165|c5_g1	**NSD**	(−)	**F**	−6.62
Fgf20b	Fibroblast growth factor 20-like/b	TR73946|c0_g1	TR56801|c1_g1	**NSD**	(−)	**F**	−3.46
Gsdf	Gonadal soma derived factor	TR72782|c0_g1	TR61049|c0_g1	**NSD**	(−)	**F**	−3.23
Rarab	Retinoid acid receptor alpha b	TR84655|c1_g1	TR63371|c2_g1	**NSD**	(−)	**F**	−2.38
Cyp11a2	Cytochrome P450, family 11, subfamily A, polypeptide 2/11alpha-hydroxylase	TR88875|c1_g5	TR61267|c0_g1	**NSD**	(−)	**F**	−2.06
Hdac11	Histone deacetylase 11	TR84940|c1_g2	TR66619|c2_g2	**NSD**	(−)	**F**	−1.44
KAT2b	Histone acetyltransferase—K(lysine) acetyltransferase 2b	TR81329|c0_g2	TR59334|c0_g1	**NSD**	(−)	**F**	−1.43
Nr3c2	Mineralocorticoid receptor	TR84031|c1_g1	TR58538|c2_g1	**NSD**	(−)	**F**	−1.06
Amhr2	Anti-Müllerian hormone receptor 2	TR90446|c1_g4	TR66380|c1_g1	**NSD**	(−)	**F**	−0.84
Fgf9	Fibroblast growth factor 9	TR91211|c1_g5	TR66253|c2_g4	**NSD**	(−)	**M**	1.86
Esr1/Er	Estrogen receptor	TR84515|c0_g1	TR68522|c4_g2	**NSD**	(−)	**M**	1.92
Pdgfaa1	Platelet-derived growth factor alpha a	TR87951|c1_g6	TR66514|c0_g1	**NSD**	(−)	**M**	2.65
Srd5a2	5α-reductase 2	(−)	TR59720|c7_g2	**(−)**		**M**	3.17

**M:** male-biased, **F:** female-biased, **NSD:** not significantly different.

### Targeted search of candidates genes for sex determination/differentiation

To identify genes that play a role in reproduction and more specifically in sex determination and differentiation of fish, we searched the annotated brain and gonad datasets for these GO categories and checked if they were differentially expressed. In particular, DE genes related to broad terms of biological processes, such as “*reproduction*” and “*reproductive process*” were searched in gonads and brains of both sparids. A large number of genes related to “*reproduction*” and a few to “*reproductive process*” were found in the gonads of both species. In the common pandora, 492 female-biased and 717 male-biased genes were found under the “*reproduction*” GO term, while thirteen female-biased and eleven male-biased genes were found under the “*reproductive process*” GO term. In the red porgy, 467 female-biased and 430 male-biased genes belonging to “*reproduction*” and nine female-biased and twelve male-biased genes belonging to “*reproductive process*” were detected. The same search in brains retrieved 246 “*reproduction*” related genes expressed in the common pandora, of which 153 were male-biased and 93 female-biased. Only four male-biased “*reproductive process*” related genes were found to be significantly expressed in common pandora brains. In the red porgy, 22 male-biased and just three female-biased “*reproduction*” related genes were found and no “*reproductive process*” related genes were among the DE genes. Further data mining, focusing on more specific GO terms, revealed nine loci in common pandora and five loci in red porgy containing the GO term “*sex determination*” and displayed significant differential expression in male and female gonads. A search for loci implicated in “*sex differentiation*” with differential expression in male and female gonads retrieved fourteen loci for common pandora, while 22 were found for red porgy. In brains, only one “*sex determination*” related and two “*sex differentiation*” related loci were found to be differentially expressed in the common pandora, while no differentially expressed loci for these GO terms were found in red porgy (Supplementary Excel Tables SET5–7). Finally, several genes expressed in male brains characterized with the GO term “*social behavior*” enriched in the red porgy and “*inter-male aggressive behavior*” and “*male courtship behavior*” in common pandora (Supplementary Excel Table SET8).

## Discussion

The present study investigated sex-specific gene-expression patterns in gonads and brains of two protogynous hermaphrodites belonging to the Sparidae family, the common pandora and the red porgy. This is the first comparative transcriptomic analysis of two species displaying the same reproductive mode, focusing on genes that are differentially expressed between sexes to pinpoint a common network that forms and/or maintains the male and female phenotype. In both species, male and female brains showed far fewer differences in expression compared to the gonads. Male and female brains were clustered together, forming a dense cluster. Quite the opposite was observed for the gonads, which differ greatly between the two sexes. Testes and ovaries were well separated and there was great variance between the formed groups (Figs 3, 4 and Supplementary Figure SF5).

### Minor sex differences indicated a more homogeneous and sexually plastic brain

Our study revealed subtle differences in expression between the sexes in the brain of the two protogynous species. This seems to be a common pattern in tissue-based studies, where sex-biased expression tended to be highest in the gonads compared to other tissues^56^. A low degree of differential expression in the brain has also been reported in other fish studies^35,39,57^. Nevertheless, in the current study we examined the brain as a whole, whereas it has been recognized that sex-specific differences in fish are noticeable only when comparing certain regions of the brain^58^. Despite the overall limited differences observed for brains compared to the gonads, common pandora male and female brains demonstrated a greater divergence than those of the red porgy, with many more genes differentially expressed between the sexes. Moreover, the direction of expression is female-biased in the common pandora, in contrast with the male-biased pattern observed here in the red porgy, but also in the sparid sharpsnout seabream^35^ and other fish examined^38,57^. However, these results were in line with the expression pattern observed in protogynous clownfish brain^39^.

A closer look into the specific genes that were up-regulated in male or female brains revealed numerous candidates with a well-known role in sex differentiation in other species. Zona pellucida (ZP) genes that showed a sex-biased expression in both brains and gonads were over-expressed in female brains. Zona pellucida proteins are glycoproteins of the fish chorion that are encoded by multiple gene families. In humans and mice, but also in some teleosts^59–61^, they are expressed in the ovary. In addition, several factors of the *Sox* gene family, as well as several members of the Forkhead box transcription factor (*Fox*) family were detected, which might play important roles in the brain of these protogynous fish. The comparative analysis revealed commonly over-expressed genes in the male brains of both species, pinpointing candidates that might make a significant contribution to the expression of the male character of the brain in protogynous sparids. Among these, the neurobeachin (*Nbea*) is required in synaptic function, regulating neurotransmitter receptor trafficking to synapses^62^. A male over-expression shared between species also displayed the *PRKCE* (Protein kinase C epsilon type) and *EphA4* (*Ephrin type-A receptor 4*). The first is implicated in platelet activation and positive regulation of the mitogen-activated protein kinase (MAPK) cascade, while the second is a receptor tyrosine kinase implicated in the ephrin receptor signaling pathway and positive regulation of Jun kinase activity. The role of such kinases in mammalian sex determination has been reported^63^, while mice studies underline the role of the phosphorylated form of MAPK (pMAPK) as a marker of neuronal activity in a reproductive context^64^.

In the common pandora and red porgy brains we also found the early growth response factor 1 (*Egr1*), the early growth response factor 4 (*Egr4*), as well as *Fos* (Proto-oncogene c-Fos) and all had a male-biased expression profile. The *Fos* gene, which encodes a nuclear phosphoprotein, reflects immediate neural activity, whereas *Egr1* is believed to regulate later-acting genes, such as gonadotropin-releasing hormone 1 (GnRH1), a primary signal essential for reproduction^65^. The *Egr1 and c-Fos*, both characterized as immediate-early genes, are related to changes in brain activity and genomic responses to social cues, behavior and mating information^66^.

Overall, in the brain of both protogynous species there were some sex-specific expression differences, although it is not known whether these differences mediate sexually dimorphic events. The more “homogeneous” transcriptome profile of the brain, initially perceived by the lack of clustering in male and female brain, became more apparent in GO term enrichment analysis, where GO terms related to female development and differentiation were over-represented in male brains and vice-versa. Sex differences in the brain, even in the human brain, are still poorly defined. As such, the issue of “brain sex differentiation” remains a controversial subject of research, especially in fish. Furthermore, the well-established phenomenon in mammals of fetal or neonatal brain exposure to sex hormones producing irreversible differences of brain structure and function, appears not to be the case in fish. Instead, fish brains are not irreversibly sexualized, conserving properties of the embryonic mammalian brain, such as high neurogenic activity, throughout life^67^. The notion that fish exhibit much more plasticity with regard to brain sex differentiation has been strengthened by the findings of the present study.

### Major sex differences in gonadal gene expression patterns

The gonads is the tissue that differs the most between sexes^68^. Despite the lability of gonadal tissue in sex-changers and the fact that testes and ovaries share a common precursor, they clearly represent two distinct types of tissue upon the establishment of sex. The functional divergence of the two gonadal types is reflected in their transcriptomic profiles, in terms of the number of genes differentially expressed, as well as the expression magnitude (*i.e*. fold-change differences). The observation of almost double the number of up-regulated genes in males compared to females indicates a male-biased expression tendency. More and more transcriptomic studies dealing with sex-specific expression differences in teleosts have pointed out male-biased over-expression tendencies^16,35,38,39,57,69,70^. In zebrafish, a study exclusively addressed the “male sex drive” hypothesis, originally proposed by Singh and Kulathinal^71^, suggesting that male–biased evolutionary pressures might have resulted in a “transcriptome masculinization” of the species. The present study, with the two protogynous hermaphrodites, along with the previous one on rudimentary sharpsnout seabream^35^, agree with a skewed ratio of male up-regulated versus female up-regulated transcripts in gonads, in favor of the first. In cases such as the protogynous hermaphrodites studied here, with no signs of heterogametic chromosomes, such a tendency may explain a great deal about the formation and maintenance of the male and female phenotypes.

### Expression profile of important molecular players in protogynous hermaphrodite gonads

Studies on vertebrates and other fish species have brought to light several candidate genes with proven or suspected relationship in sex determination and differentiation. As there is scanty literature-based information regarding the sex-related genetic cascade in these protogynous species, we compiled a list of well-studied sex-biased genes and searched for their expression patterns in the gonadal transcriptomes of both sparids. Despite the plethora of different sexual systems in fish, there is some evidence of conservation of the genetic cascade, with its “basic” components (molecular players) found repeatedly, not only among fishes but also generally in vertebrates^72,73^. However, the specific role and place for each component of this at least partially conserved “crew” is not prominent and might change in different species and/or sexual systems^57,74,75^.

**Figure 6:**
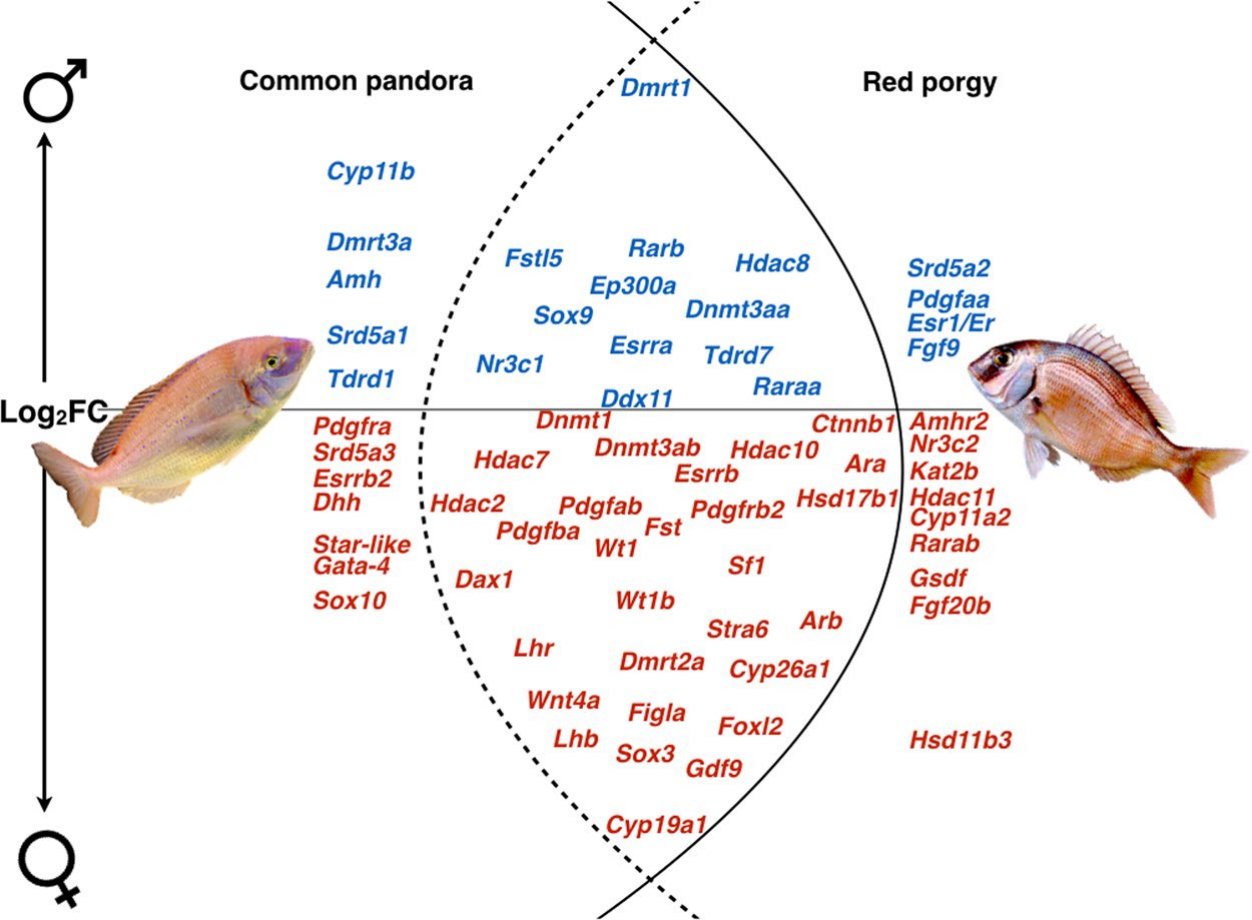
Comparative view of known sex-related genes, commonly (intersection) and uniquely expressed in common pandora (left) and red porgy (right). Above and below the central line are the male-specific (Blue) and the female-specific (Red) genes. The Y-axis serves as an indication of expression magnitude (in terms of Log2Fold-Change differences). The commonly expressed genes (intersection) are placed according to their Log2Fold-Change values in common pandora and had an analogous expression magnitude in red porgy. The genes uniquely expressed in each species are placed according to their expression values in that particular species.

Based on the literature, we profiled the expression of known candidate (Fig. 6) molecular-players/genes establishing the common female (*Cyp19a1, Sox3, Foxl2, Figla, Wnt4a, Gdf9, Fst, Lhb, Ara, Arb*, *etc.)* and male identity (*Dmrt1, Sox9, Dnmt3aa, Rarb, Raraa, Hdac8*, etc.) of the gonads of these protogynous sparids. Additionally, we focused on those contributing in species-specific manner either to female (*Gata-4, Sox10, Amhr2, Gsdf*, Srd5a3 etc.) or to male (*Amh, Dmrt3a, Srd5a1, Srd5a2*, etc.) characters. Starting with the formation and maintenance of the female phenotype, some genes over-expressed, showed high Log_2_ fold changes in the ovaries of both sparids. The *Figla* gene that appears to have a decisive role in the female phenotype of both species is such an example. This transcription factor up-regulates ZP genes, which code for products used to build the vitelline envelope surrounding eggs in teleosts (zona matrix)^76–78^. Moreover, it has been suggested that the *Figla* factor displays an anti-parallel direction of expression to *Dmrt1* during the transition from male to female in a protandrous^79^ and vice versa in a protogynous teleosts^80^. The same expression pattern was showed for *Foxl2*, a prominent marker of ovarian development across vertebrates^81^. The *Foxl2* has been reported as a female-biased gene in several gonochoristic^82–85^ and, recently, in some hermaphroditic species^38,39^.

The gene of ovarian aromatase is probably the one with the most protagonistic role in gonadal fate. Together with estrogens it has been hypothesized to control not only the ovarian, but also the testicular differentiation. When it is over-expressed it appears to trigger and maintain ovarian differentiation and once its expression reduces it appears to allow for testicular differentiation to take place^86^. Here, a strong female-biased expression of gonadal aromatase gene (*Cyp19a1a*) was observed in both species. In fish, *Cyp19a1a* has an essential role in ovarian differentiation. Its expression was not only reported in gonochorists^75,87–89^, but also in other hermaphrodite fish^39,90–94^ which repeatedly highlights its involvement with the gonadal sex differentiation and sex change. It has been also suggested that the expression of this conserved and well-characterized protein is controlled not only by a “classical” transcriptional activator such as *Foxl2*, but also by epigenetic modifications^95^. Gonadal aromatase gene and its regulation through methylation modifications has also been involved in thermo-sensitive sex differentiation systems in fish^92,96^. Thus, changes in methylation levels of the *Cyp19a1a* promoter might be an additional control factor in sex-changing species^95^. The precise role of aromatase in the sex determination/differentiation systems of these species awaits further and deeper studies. The *Cyp19a1a* promoter contains binding sites for *Wilms tumor 1 protein* (*Wt1*)^86^. In mammals, there is only one *Wt1* gene critical for development of the urogenital system, whilst in teleosts two *Wt1* (*Wt1a, Wt1b*) genes have been identified and both are over-expressed in ovaries^97^.

The Luteinizing hormone (*Lh*) and its receptor (*Lhr)* showed a strong female over-expression in both species examined here. According to studies in medaka, both genes are involved in fish ovulation^98^. A common female-biased expression also displayed the fushi tarazu factor-1 (*Ftz-F1*) or steroidogenic factor-1 (*Sf-1*). Interestingly, the opposite has been described in two other hermaphrodites^38,39^ and in some well-known gonochorists^99,100^, where *Sf-1* gene has been reported as a male-biased gene. It has been hypothesized that *Sf-1/Ftz-F1* along with some additional partners, such as *Foxl2* and *cAMP*, co-regulate the transcription of aromatase, as the later contains binding sites for *Sf-1* genes^86^. Two members of the Wnt/b-catenin pathway, *Wnt4* (wingless-type MMTV integration site family, member 4) and *Ctnnb1* (Catenin, beta 1) and *Fst* (follistatin) well recognized markers of ovarian differentiation linked to this pathway, all seem to contribute to the female identity of the gonads in both sparids studied here.

Three factors of the retinoic acid (RA) signaling pathway were found in the transcriptome of both species and of those three factors *Cyp26a1* over-expressed in their ovaries of both sparids. The role of this pathway in sex determination/differentiation and particularly in ovarian differentiation^101–103^ is further supported by respective findings among ovary-enriched GO terms. Recently, a RA homeostatic mechanism stimulated by retinoic acid gene 8 (*Stra8*) via an independent pathway has been shown to implicate that *Cyp26a1* and *Aldh1a2* mediate the meiotic entry of germ cells in Nile tilapia^102^. While *Stra8* was absent from the transcriptomes of the studied sparids, *Stra6* was present and over-expressed in the ovaries of both species. A search for genes encoding RA receptors (*Raraa*, *Rarab* and *Rarb*) revealed that all three exhibited sex-biased expression profiles. The first two were commonly male-biased, while *Rarb* was over-expressed in red porgy ovaries.

Four members of the TGF-b superfamily (*Gdf9, Gsdf, Amh, Amhr2*) were found in both species’ transcriptomes and displayed sex-biased expression. In particular, growth differentiation factor-9 (*Gdf9*), which is required for ovarian folliculogenesis^104^ was up-regulated in the ovaries of both species. Our findings are in line with those of another protogynous fish, the bluehead wrasse^38^. The gonadal soma derived factor (*Gsdf*), a gene probably unique to teleosts and recently identified as the male-specific master sex-determining gene in *Oryzias luzonensis*^10^ was uniquely over-expressed in the ovaries of red porgy, possibly indicating a different role for the *Gsdf* gene in this protogynous species. A well-known member of the TGF-b family, the anti-Mullerian hormone (*Amh*) seems to contribute to the common pandora male phenotype, while it showed no sex-biased differences in red porgy. Interestingly, its receptor, *Amhr2*, is female-biased in the later species. The *Amh* and *Amhr2* are known master SD genes in the Patagonian pejerrey *Odontesthes hatcheri*^11^ and in the tiger pufferfish *Takifugu rubripes*^12^, respectively. The *Amh* role in teleost sexual differentiation, which lack Müllerian ducts, is not clear, though it has been proposed that proliferation and differentiation of spermatogonia are the possible common functions of this gene among vertebrates^105,106^. The same gene has also been found among the male-specific genes in some gonochoristic^57,107^ and hermaphroditic^35,39^ fish and it is involved in the sex change of the protandrous black porgy^90^.

Two major components of the Sox genes^108^ (*Sox3* and *Sox9*) were found among the commonly expressed gonadal genes between the two protogynous species. The present study demonstrated that *Sox3* has a high female-specific expression in both common pandora and red porgy. In mammals, *Sox3* is implicated in the induction of testis formation^109^ and spermatogenesis^110^. However, its role in fish sex determination and differentiation is still ambiguous as in some studies it is linked to the male phenotype^14,111^, while in others to the female^38,57,112^. The other Sox gene, *Sox9*, is a male-biased gene in both species. The direction of expression of *Sox9* agrees with the male-specific role of this gene in mammals^113^. Nevertheless, *Sox9* has been reported to have a male-biased expression in rudimentary sharpsnout seabream^35^, the protogynous bluehead wrasse^38^ and the protandrous clownfish^39^. On the contrary, in the medaka *Sox9* is expressed in ovaries^114^. Interestingly, in zebrafish *Sox9* has two homologues, one expressed in oocytes and the other in testicular Sertoli cells^115^. Taken together, the results suggest that *Sox3* and *Sox9* are important in the common pandora and red porgy female and male phenotype formation respectively and suggest both genes as handy molecular players that could contribute to either sex side, in teleosts.

Despite major differences in the genetic control of sexual development in different organisms, *Dmrt* genes are actively involved in sex-specific differentiation in vertebrates, invertebrates and all teleosts that have been studied so far^116–118^. A family member, *Dmrt2/Dmrt2a* which is linked to neurogenesis^119^ and left-right patterning^120^ during embryonic development, displayed a female-biased expression shared between species. This agrees with the patterns observed in the Atlantic cod^121^ and four cichlids species^57^. On the other hand, the most studied member of this gene family, *Dmrt1*, was highly up-regulated in the testes of both protogynous species studied. Hence, the results of this study indicate that *Dmrt1* together with *Sox9* could play a fundamental role in expressing and preserving the male character in gonad. The role of *Dmrt1* in sex determination and spermatogenesis in vertebrates and in testicular development and maintenance in teleosts is well established^117^. In line with the current study results, characteristic male-biased expression of *Dmrt1* has been reported in numerous gonochoristic^99,122–124^ and other hermaphrodite teleosts^33,35,38,39,91^. *Dmrt3*/*Dmrt3a*, another male-specific gene in general^125^, appears to contribute to male phenotype of common pandora, as it was strongly expressed in their testes, but it was absent from the red porgy transcriptome.

Another interesting finding from our study was the expression profiles of androgen and estrogen receptor genes (*Ara*, *Arb*, *Er/Esr1* and *Esr2b*), as well as estrogen-related receptors (*Esrra* and *Esrrb*) in the gonadal transcriptomes of both species. Surprisingly, both androgen receptors were up-regulated in the ovaries of both species and *Esrra* displayed a common male up-regulation. Although it is still unclear how the endocrine mechanism works to control sex change, steroid hormones are believed to regulate the process^126,127^.

Finally, genes of epigenetic maintainers, such as the DNA-modifying enzymes DNA methyltransferases (*Dnmt3aa, Dnmt1, Dnmt3ab*) and histone tail-modifying and de-modifying enzymes, such as histone acetyl transferases (*Ep300a, Kat2b*) or histone deacetylases (*Hdac8, Hdac2, Hdac10, Hdac11*) were found in transcriptome of both protogynous sparids and showed a sex-biased expression. Further, enrichment analysis revealed genes involved in epigenetic related mechanisms, supporting their implication in sex determination/differentiation and apparently sex maintenance. The relationship of the epigenetic mechanisms to sex determination and differentiation in vertebrates has been clearly stated and these mechanisms are considered to be the link between environmentally controlled gene expression and sex reversal^95^.

## Conclusions

In this study, we sequenced the total mRNA from the gonads and brains of both sexes in two protogynous hermaphrodite sparids, the common pandora and the red porgy. This approach permitted us to obtain a global view of sex-specific expression patterns, as well as it allowed us to compare sex-biased gene expression patterns within teleosts having the same reproductive mode. Focusing on the pathways and genes implicated in sex determination/differentiation, we tried to unveil the molecular pathway by which a protogynous hermaphrodite fish develops a masculine or a feminine character. We found minor sex-related expression differences indicating a more homogeneous and sexually plastic brain, whereas there was a plethora of sex biased gene expression in the gonads. This indicates functional divergence of the transformed tissue either to male or female character, even though both types of gonadal tissue originated from the same precursor. We observed the implicated pathways behind sex-biased expression and saw the recruitment of known sex-related genes either to male or female type of gonads in these fish. Taken together, most of the studied genes form part of the cascade of sex determination, differentiation and reproduction across teleosts. In this study, we focused on genes that are active when sex is maintained (sex-maintainers). Comparing related species with the same reproductive style, we saw different combinations of genes with conserved sex-linked roles and some handy molecular players, in a partially conserved network formulating the male and female phenotype. Finally, this analysis lays the ground for understanding the complex process of sex differentiation in these protogynous sparids and makes future comparative analyses of fish with alternative reproductive modes feasible. Future work in gene expression data during sex-reversal will uncover which genes are triggered to block female path and start to transform the gonadal tissue in the opposite direction.

## Methods

### Sample collection

Animal care was carried out according to the “Guidelines for the treatment of animals in behavioural research and teaching”^128^. The fish used in the study were maintained in registered and approved facilities for the maintenance and carrying of animal experiments and were reared and sampled according to the guidelines of the Directive 2010/63/EU for the protection of animals used for experimental and other scientific purposes (Official Journal L276/33)^129^ and the guidelines of Metcalfe and Craig (2011)^130^. In addition, the experimental sampling protocol was approved by the IMBBC’s aquaculture department committee and all methods were carried out in accordance with relevant guidelines and regulations approved by the Ministry of Rural Development and Food and the Regional Directorate of Veterinary Medicine for certified experimental installations (EL 91-BIO-04) and experimental animal breeding (AQUALABS, EL 91-BIO-03). Members of the laboratory include certified technicians by the Federation for Laboratory Animal Science Associations (FELASA).

The fish used for the experiments were either wild fish caught alive, or broodstock fish of wild origin in the Institute of Marine Biology, Biotechnology and Aquaculture (HCMR) (Supplementary Excel Tables SET9). In both cases, mature fish were euthanized using the commercially available slaughter method for marine fish (*i.e*. immersion in ice-slurry) and the brain and gonads were dissected immediately. Only one of the two gonadal lobes was kept and stored until further processing. The fish were examined macroscopically for sexual maturation, based on the presence of releasable sperm for males or the presence of vitellogenic oocytes for females and/or visual inspection of the gonad morphology. For red porgy, nine brain and nine gonad tissues were sampled from five female and four male individuals, while for common pandora ten brain and ten gonad tissues were sampled from five females and five males. In addition, for each species a pool of several hundred larvae at the “hatching” stage (1 days post hatch) were sampled to increase the possibility of capturing the majority of the species transcripts. All tissues and larvae were collected in a sterile and RNase-free way and stored immediately in RNAlater (Applied Biosystems, Foster City, CA, USA). Tissues for RNA extraction were stored at 4 °C overnight according to the manufacturer’s recommendations and then transferred to −80 °C until further processing.

### RNA extraction and sequencing

To reduce biases regarding different expression in different parts of the same organ, we homogenized the whole brain, one lobe of testis and one lobe of ovaries. All tissue samples, including larvae, were ground with liquid nitrogen using pestle and mortar, homogenized in TRIzol^®^ reagent (Invitrogen) and total RNA was extracted from the TRIzol^®^ homogenate according to the manufacturer’s instructions. The quantity of the isolated RNA was measured spectrophotometrically with NanoDrop^®^ ND-1000 (Thermo Scientific), while its quality was tested on an agarose gel (electrophoresis in 1.5% w/v) and further on an Agilent Technologies 2100 Bioanalyzer (Agilent Technologies). The majority of the samples had an RNA Integrity Number (RIN) value higher than 8. This was not the case for one testes from common pandora and it was excluded from the further analysis. In contrast to their high quality agarose image, all ovaries showed lower RIN values. The low RIN values obtained from ovary samples could be due to the abundance of low molecular weight 5 S RNA expression. In a previous study of sharpsnout sea bream^35^ where the RNA extractions were carried out with a commercial kit and the female gonads were in an early maturing stage, the 5 S rRNA was in such abundance that it masked the 18 S and 28 S rRNA peaks thus hampering the correct estimation and calculation of RNA integrity numbers (RIN). This low molecular weight band of 120 bp, belonging to the smallest rRNA molecule, was also detected in the ovaries and intersex gonads of thicklip grey mullet *Chelon labrosus*, where the role of 5 S rRNA in fish sex differentiation and its potentiality as a sex marker in fish gonads has been thoroughly described^131^. This type of RNA profile was also observed in female gonads of bluehead wrasse^38^ and very recently a study described the use of ribosome RNA profile not only as a sex identifier but also as an indicator of ovarian developmental stage^132^. Finally together with two larval samples, 39 samples were used for mRNA paired-end library construction with the Illumina TruSeq^TM^ RNA Sample Preparation Kits v2 following the manufacturer’s protocol (Poly-A mRNA isolation with oligo-dT beads, mRNA fragmentation, followed by transcription into first-strand cDNA using reverse transcriptase and random hexamer primers) and sequenced as 100 bp paired reads in three lanes (1,5 lanes per species) of a HiSeq. 2500^TM^ following the protocols of Illumina Inc. (San Diego, CA).

### Read pre-processing

All computations were performed at the high-performance computing bioinformatics platform of HCMR. Read quality was assessed with FastQC^133^ and subjected to quality control following a pipeline using Scythe^134^, Sickle^135^, Trimmomatic^136^ and Prinseq (prinseq-lite-0.20.3)^137^. We first used Scythe **-** A Bayesian adapter trimmer (version 0.994 BETA)^134^, which uses a Naive Bayesian approach to classify contaminant substrings in sequence reads; it considers quality information which can make it robust in picking out 3′-end adapters (which often include poor-quality bases) and we therefore ran it before any quality-based trimming (with the prior contamination rate set in 0.1 ‘-p 0.1’). Low-quality (parameters ‘pe -g -t sanger -q 20 -l 45’) nucleotide reads trimming was implemented with Sickle^135^, a sliding-window, adaptive, quality-based trimming tool for FastQ files. Using Trimmomatic^136^, some extra filtering steps and also both 5′ and 3′ adaptor removal were performed (parameters ‘PE -phred33 ILLUMINACLIP:adapter_file.fa:2:30:10 LEADING:3 TRAILING:3 SLIDINGWINDOW:4:25 MINLEN:45 CROP:99’). Finally, low- complexity sequences (threshold entropy value of 30) and poly A/T 5′ tail (minimum of 5 A/T) trimming was performed using PrinSeq^137^. Lastly, the reads of each pair of all the libraries were combined using custom Perl scripts.

### Transcriptome assembly construction

The *de novo* assemblies for red porgy and common pandora were performed using Trinity package^138^ (version 2.0.6) with default parameters (default kmer 25, min length 200 nucleotides). To increase transcriptome coverage, *de novo* assemblies were carried out on a pool of the pre-processed reads from all samples. To assess the assembled transcripts and exclude the spurious ones, we pooled all filtered reads and mapped them to the selected assemblies (Pagrus_Trinity_assembly.fasta and Pagellus_Trinity_assembly.fasta) with Bowtie2^51^ within RSEM (version 1.2.19)^139^, using the script available in trinity utilities *align_and_estimate_abundance.pl*. Putative transcripts with less than 1% of a locus reads mapped to that particular isoform (IsoPct <1) were excluded (as proposed in)^140^. The same was done for those with Fragments Per Kilobase of transcript per Million mapped read (FPKM) values less than 0.3. The choice of FPKM threshold was based on BLASTn similarity searches (*e* -value threshold 10^−10^) against European sea bass cDNA sequences (http://seabass.mpipz.mpg.de). We first contacted a blast search of the unfiltered assemblies against the European sea bass cDNA sequences and then repeated the blast and tested three differently filtered assemblies (a) FPKM_cutoff = 0.3, isopct_cutoff = 1.0, (b) FPKM_cutoff = 0.5, isopct_cutoff = 1.0 and (c) FPKM_cutoff = 1, isopct_cutoff = 1.0. To assess the assembled transcripts quality, we followed three methods: (a) pooling all the reads and mapping them to the assembly using Bowtie2^51^ and Samtools^141^ to estimate the percentage of mapped reads, (b) examining the similarity between the Trinity assembly and swissprot database using blastx search and (c) quantifying the completeness of the transcriptome assembly and determining how many of the 3,023 genes for vertebrates (OrthoDBv8 was used from BUSCO) were present in our two species transcriptomes, using BUSCO (Benchmarking Universal Single-Copy Orthologs) software^142^ that searches for highly conserved, near-universal, single copy orthologs. All BLAST^143^ searches were performed in parallel using NOBLAST program^144^.

### Transcriptomes functional annotation

For the annotation of the assembled transcripts, we conducted a BLASTx similarity search against UniProtKB/SwissProt database^52^ (*e*-value threshold 10^−5^) keeping the top 20 hits. BLASTx was done in parallel using NOBLAST^144^. For gene ontology (GO) mapping, Blast2GO^54^ was used locally to recover all the GO terms associated to the hits obtained by the blast search. After the mapping step, results were subjected to GO annotation, a process of selecting GO terms and assigning them to the query sequences for describing biological processes, molecular functions and cellular components. Gene ontology graphs were generated in “level 2” for GO plot visualization and pie charts for the whole transcriptome profiling. Further, brains and gonads of each species were analyzed separately, using in-house R scripts. The Blast2GO output file was input into the BGI WEGO program^55^ and GO annotations were plotted. The sequences were additionally annotated using InterPro^145^; a local InterProScan (version 5, InterPro release 48.0)^53^ was run in parallel (splitting the query set) on the longest open reading frame (ORF) of the contigs using a custom Perl script based on the EMBOSS program “getorf”^146^. GO terms corresponding to these InterPro domains, were merged with the already existing GO terms derived from the blastx against UniProt. The GO terms were modulated using the annotation augmentation tool ANNEX^147^ followed by GOSlim^148^. Enzyme classification (EC) codes were obtained through the direct mapping of GO terms to the corresponding enzyme codes. Sequences having EC numbers were further characterized by Kyoto Encyclopedia of Genes and Genomes (KEGG)^149^ metabolic pathway annotations using custom Perl/R scripts.

### Differential expression analysis

Differential expression analysis was performed at the “gene” (component/unigene) level by pairwise comparisons between male and female for brain and gonad samples. The *de novo* transcriptome assembly of each species served as a reference for read mapping. For the quantification, the cleaned reads of gonads and brains (not larvae) of each sample of a species were aligned to the species’ transcriptome assembly with Bowtie2^51^ and abundance was estimated with RSEM, as implemented in the trinity script *align_and_estimate_abundance.pl*. The estimated expected counts for each sample, when the sum of counts in each row >15, were extracted^150^. Loci with less than fifteen reads mapped on them, were excluded prior to differential expression analysis to improve the statistical power. Count data were imported in R (version 3.2.0) and the analysis of differential expression conducted in DESeq. 2 (version: 1.8.1)^151^, an R bioconductor package^152^. Samples of both species were grouped according to sex and expression was compared separately for brains and gonads, following the developers’ manual (false discovery rate, FDR, threshold of 0.05). Principal component analysis (PCA)^153^ and the heatmap.2 function in the gplots package^154^ were used to visualize global similarities and differences among either the brain or the gonadal samples. The global expression analysis conducted with sex and tissue being the (*design/variable/intgroup)* factors influencing the counts in a multifactor analysis. Each species global (brains and gonads) PCA plot was produced including all the genes in the filtered (sum of counts in each row >15) count matrix. Genes with an adjusted *p* value less than 0.05 were considered as significantly differentially expressed.

### Functional GO and pathway enrichment analysis

Functional-enrichment analysis was performed to identify GO terms and metabolic pathways significantly enriched in differentially expressed genes in brains and gonads, in the two sparids. In each species, male and female up-regulated gonad and brain gene sets were tested for enriched GO terms, in a comparison of female versus male (female/test-set vs male/reference-set). Particularly for gonads, we restricted the gene-set to only those with |log_2_FC| >2. We also conducted enrichment analysis for common (between species) male-biased and female-biased gene set using both ‘red porgy’ and ‘common pandora’ total datasets as reference set of the annotated transcripts. All enrichment analyses were implemented in BLAST2GO with default settings enabling for a two-tailed test and reducing the output table to the most specific terms with FDR = 0.05.

### Targeted search for sex-related candidate genes

To identify transcripts related with the GO terms “*reproduction*” (GO:0000003), “*reproductive process*” (GO:0022414), “*sex determination*” (GO:0007530) and “*sex differentiation*” (GO:0007548), both annotated transcriptomes were searched for the presence of these GO terms and the retrieved genes were checked for whether they exhibit a male or female-biased expression in brains and gonads. Genes known to be involved in sex determination/differentiation in vertebrates and other fish taxa were downloaded for tilapia, medaka and spotted gar *Lepisosteus oculatus f*rom Ensembl (release 84)^155^, resulting in a set of 114 candidate genes. To find out if orthologs of these genes were present in our species transcriptomes, we used a reciprocal BLASTn best-hit approach^156^. The potentially orthologous genes were then tested based on the differences in their expression patterns between testes and ovaries of both species by using the output files of DeSeq. 2. Those that were differentially expressed in both species or were species-specific were identified.

### Availability of supporting data

The datasets generated and/or analysed during the current study are available from the corresponding author upon request. Raw sequence reads can be found in the SRA database under BioProject ID: PRJNA395994.

## Electronic supplementary material

Supplementary Information 

Supplementary Dataset 1

Supplementary Dataset 2

Supplementary Dataset 3

## References

[1] Mittwoch U (2005). Sex determination in mythology and history. Arq. Bras. Endocrinol. Metabol..

[2] Heule C, Salzburger W, Böhne A (2014). Genetics of Sexual Development: An Evolutionary Playground for Fish. Genetics.

[3] Desjardins JK, Fernald RD (2009). Fish sex: why so diverse?. Curr. Opin. Neurobiol..

[4] Tobias U (2011). & Heikki Helanterä. From the Origin of Sex-Determining Factors to the Evolution of Sex-Determining Systems. Q. Rev. Biol..

[5] Yoshimoto S (2008). A W-linked DM-domain gene, DM-W, participates in primary ovary development in Xenopus laevis. Proc. Natl. Acad. Sci. USA.

[6] Sinclair AH (1990). A gene from the human sex-determining region encodes a protein with homology to a conserved DNA-binding motif. Nature.

[7] Koopman P, Gubbay J, Vivian N, Goodfellow P, Lovell-Badge R (1991). Male development of chromosomally female mice transgenic for Sry. Nature.

[8] Smith CA (2009). The avian Z-linked gene DMRT1 is required for male sex determination in the chicken. Nature.

[9] Matsuda, M. *et al*. DMY is a Y-specific DM-domain gene required for male development in the medaka fish. *Nature* **417** (2002). 10.1038/nature751 12037570

[10] Myosho T (2012). Tracing the Emergence of a Novel Sex-Determining Gene in Medaka, Oryzias luzonensis. Genetics.

[11] Hattori RS (2012). A Y-linked anti-Müllerian hormone duplication takes over a critical role in sex determination. Proc. Natl. Acad. Sci. USA.

[12] Kamiya T (2012). A Trans-Species Missense SNP in Amhr2 Is Associated with Sex Determination in the Tiger Pufferfish, Takifugu rubripes (Fugu). PLoS Genet.

[13] Yano A (2012). An Immune-Related Gene Evolved into the Master Sex-Determining Gene in Rainbow Trout, Oncorhynchus mykiss. Curr. Biol..

[14] Takehana Y (2014). Co-option of Sox3 as the male-determining factor on the Y chromosome in the fish Oryzias dancena. Nat. Commun..

[15] Chen S (2014). Whole-genome sequence of a flatfish provides insights into ZW sex chromosome evolution and adaptation to a benthic lifestyle. Nat Genet.

[16] Tao W (2013). Characterization of gonadal transcriptomes from Nile tilapia (Oreochromis niloticus) reveals differentially expressed genes. PLoS One.

[17] Nicol B, Guiguen Y (2011). Expression Profiling of Wnt Signaling Genes during Gonadal Differentiation and Gametogenesis in Rainbow Trout. Sex. Dev..

[18] Kobayashi T, Kajiura-Kobayashi H, Guan G, Nagahama Y (2008). Sexual dimorphic expression of DMRT1 and Sox9a during gonadal differentiation and hormone-induced sex reversal in the teleost fish Nile tilapia (Oreochromis niloticus). Dev. Dyn..

[19] Kovács B, Egedi S, Bártfai R, Orbán L (2000). Male-specific DNA markers from African catfish (Clarias gariepinus). Genetica.

[20] Ezaz MT (2004). Isolation and Physical Mapping of Sex-Linked AFLP Markers in Nile Tilapia (Oreochromis niloticus L.). Mar. Biotechnol..

[21] Felip A, Young WP, Wheeler PA, Thorgaard GH (2005). An AFLP-based approach for the identification of sex-linked markers in rainbow trout (Oncorhynchus mykiss). Aquaculture.

[22] Martínez P (2009). Identification of the Major Sex-Determining Region of Turbot (Scophthalmus maximus). Genetics.

[23] Shao C-W (2010). Construction of Two BAC Libraries from Half-Smooth Tongue Sole Cynoglossus semilaevis and Identification of Clones Containing Candidate Sex-DeterminationGenes. Mar. Biotechnol..

[24] Phillips RB, Park LK, Naish KA (2013). Assignment of Chinook Salmon (Oncorhynchus tshawytscha) Linkage Groups to Specific Chromosomes Reveals a Karyotype with Multiple Rearrangements of the Chromosome Arms of Rainbow Trout (Oncorhynchus mykiss). G3 Genes|Genomes|Genetics.

[25] Iturra P, Lam N, dela Fuente M, Vergara N, Medrano JF (2001). Characterization of sex chromosomes in rainbow trout and coho salmon using fluorescence *in situ* hybridization (FISH). Genetica.

[26] Larson WA, McKinney GJ, Seeb JE, Seeb LW (2016). Identification and Characterization of Sex-Associated Loci in Sockeye Salmon Using Genotyping-by-Sequencing and Comparison with a Sex-Determining Assay Based on the sdY Gene. J. Hered..

[27] Ross JA, Urton JR, Boland J, Shapiro MD, Peichel CL (2009). Turnover of Sex Chromosomes in the Stickleback Fishes (Gasterosteidae). PLoS Genet..

[28] Henning F, Moysés CB, Calcagnotto D, Meyer A, de Almeida-Toledo LF (2011). Independent fusions and recent origins of sex chromosomes in the evolution and diversification of glass knife fishes (Eigenmannia). Heredity (Edinb)..

[29] Palaiokostas C (2015). A new SNP-based vision of the genetics of sex determination in European sea bass (Dicentrarchus labrax). Genet. Sel. Evol..

[30] Mylonas, C. C., Zohar, Y., Pankhurst, N. & Kagawa, H. Reproduction and broodstock management. in *Sparidae: Biology and Aquaculture of Gilthead Sea* Bream *and Other Species* (eds Pavlidis, M. A. & Mylonas, C. C.) 95–131 (Blackwell Publishing Ltd, 2011).

[31] Buxton CD, Garratt PA (1990). Alternative reproductive styles in seabreams (Pisces: Sparidae). Environ. Biol. Fishes.

[32] Wu G-C, Chang C-F (2013). The switch of secondary sex determination in protandrous black porgy, Acanthopagrus schlegeli. Fish Physiol. Biochem..

[33] He CL (2003). Differential Dmrt1 transcripts in gonads of the protandrous black porgy, Acanthopagrus schlegeli. Cytogenet. Genome Res..

[34] Loukovitis D (2012). Quantitative trait loci for body growth and sex determination in the hermaphrodite teleost fish Sparus aurata L. Anim Genet.

[35] Manousaki T (2014). The sex-specific transcriptome of the hermaphrodite sparid sharpsnout seabream (Diplodus puntazzo). BMC Genomics.

[36] Erisman, B. E., Petersen, C. W., Hastings, P. A. & Warner, R. R. Phylogenetic Perspectives on the Evolution of Functional Hermaphroditism in Teleost Fishes. *Integr. Comp. Biol*. 10.1093/icb/ict077 (2013). 10.1093/icb/ict077 23817661

[37] Ravi P, Jiang J, Liew WC, Orbán L (2014). Small-scale transcriptomics reveals differences among gonadal stages in Asian seabass (Lates calcarifer). Reprod. Biol. Endocrinol..

[38] Liu H (2015). Large-scale transcriptome sequencing reveals novel expression patterns for key sex-related genes in a sex-changing fish. Biol. Sex Differ..

[39] Casas L (2016). Sex Change in Clownfish: Molecular Insights from Transcriptome Analysis. Sci. Rep..

[40] Basurco, B., Lovatelli, A. & Garcia, B. Current status of Sparidae aquaculture. in *Sparidae: Biology and Aquaculture of Gilthead Sea Bream and Other Specie*s(eds Pavlidis, M. A. & Mylonas, C. C.)1–50 (Blackwell Publishing Ltd).

[41] Bauchot, M. L. & Hureau, J. C. *Sparidae*. *Fishes of the North-eastern Atlantic and the Mediterranean* **2** (1986).

[42] Shapiro DY (1992). Plasticity of gonadal development and protandry in fishes. J. Exp. Zool..

[43] Warner RR, Robertson DR, Leigh EG (1975). Sex change and sexual selection. Science (80-)..

[44] Cataudella S, Riz PP, Sola L (1980). A chromosome study of eight Mediterranean species of Sparidae (pisces, perciformes). Genetica.

[45] Manousaki T (2016). Exploring a Nonmodel Teleost Genome Through RAD Sequencing—Linkage Mapping in Common Pandora, Pagellus erythrinus and Comparative Genomic Analysis. . G3 Genes|Genomes|Genetics.

[46] Avise JC, Mank JE (2009). Evolutionary Perspectives on Hermaphroditism in Fishes. Sex. Dev..

[47] Godwin J (2010). Neuroendocrinology of sexual plasticity in teleost fishes. Front. Neuroendocrinol..

[48] Larson, E. T. Neuroendocrine regulation in sex-changing fishes. in *Hormones and Reproduction of Vertebrates:* Fishes (eds Norris, D. O. & Lopez, K. H.) **1**, 149–168 (Elsevier, 2011).

[49] Wang, Z., Gerstein, M. & Snyder, M. RNA-Seq: a revolutionary tool for transcriptomics. *Nat Rev Genet* **10** (2009). 10.1038/nrg2484 PMC2949280 19015660

[50] Wilson CA, Davies DC (2007). The control of sexual differentiation of the reproductive system and brain. Reproduction.

[51] Langmead, B. & Salzberg, S. L. Fast gapped-read alignment with Bowtie 2. *Nat Methods* **9** (2012). 10.1038/nmeth.1923 PMC3322381 22388286

[52] Consortium TU (2015). UniProt: a hub for protein information. Nucleic Acids Res..

[53] Quevillon E (2005). InterProScan: protein domains identifier. Nucleic Acids Res..

[54] Conesa A (2005). Blast2GO: a universal tool for annotation, visualization and analysis in functional genomics research. Bioinformatics.

[55] Ye J (2006). WEGO: a web tool for plotting GO annotations. Nucleic Acids Res..

[56] Grath, S. & Parsch, J. Sex-Biased Gene Expression. *Annu. Rev. Genet*. 10.1146/annurev-genet-120215-035429 (2015). 10.1146/annurev-genet-120215-035429 27574843

[57] Böhne A, Sengstag T, Salzburger W (2014). Comparative Transcriptomics in East African Cichlids Reveals Sex- and Species-Specific Expression and New Candidates for Sex Differentiation in Fishes. Genome Biol Evol.

[58] O’Connell LA, Ding JH, Hofmann HA (2013). Sex differences and similarities in the neuroendocrine regulation of social behavior in an African cichlid fish. Horm. Behav..

[59] Kanamori A, Naruse K, Mitani H, Shima A, Hori H (2003). Genomic organization of ZP domain containing egg envelope genes in medaka (Oryzias latipes). Gene.

[60] Modig C (2006). Molecular Characterization and Expression Pattern of Zona Pellucida Proteins in Gilthead Seabream (Sparus aurata)1. Biol. Reprod..

[61] Hyllner SJ, Westerlund L, Olsson PE, Schopen A (2001). Cloning of rainbow trout egg envelope proteins: members of a unique group of structural proteins. Biol. Reprod..

[62] Nair R (2013). Neurobeachin regulates neurotransmitter receptor trafficking to synapses. J. Cell Biol..

[63] Windley SP, Wilhelm D (2015). Signaling Pathways Involved in Mammalian Sex Determination and Gonad Development. Sex. Dev..

[64] Taziaux M, Keller M, Balthazart J, Bakker J (2011). Rapid activation of phosphorylated MAPK after sexual stimulation in male mice. Neuroreport.

[65] Burmeister SS, Fernald RD (2005). Evolutionary conservation of the egr-1 immediate-early gene response in a teleost. J. Comp. Neurol..

[66] Desjardins JK, Klausner JQ, Fernald RD (2010). Female genomic response to mate information. Proc. Natl. Acad. Sci. USA.

[67] Le Page Y (2010). Aromatase, brain sexualization and plasticity: the fish paradigm. Eur. J. Neurosci..

[68] Mank JE, Hultin-Rosenberg L, Webster MT, Ellegren H (2008). The unique genomic properties of sex-biased genes: Insights from avian microarray data. BMC Genomics.

[69] Xu D (2016). The testis and ovary transcriptomes of the rock bream (Oplegnathus fasciatus): A bony fish with a unique neo Y chromosome. Genomics Data.

[70] Machado, M. P., Matos, I., Grosso, A. R., Schartl, M. & Coelho, M. M. Non-canonical expression patterns and evolutionary rates of sex-biased genes in a seasonal fish. *Mol. Reprod. Dev*. n/a-n/a 10.1002/mrd.22752 (2016). 10.1002/mrd.22752 27770608

[71] Singh RS, Kulathinal RJ (2005). Male sex drive and the masculinization of the genome. BioEssays.

[72] Volff JN, Nanda I, Schmid M, Schartl M (2007). Governing Sex Determination in Fish: Regulatory Putsches and Ephemeral Dictators. Sex. Dev..

[73] Smith C, Wootton RJ (2016). The remarkable reproductive diversity of teleost fishes. Fish Fish..

[74] Herpin A (2013). Divergent Expression Regulation of Gonad Development Genes in Medaka Shows Incomplete Conservation of the Downstream Regulatory Network of Vertebrate Sex Determination. Mol. Biol. Evol..

[75] Böhne A, Heule C, Boileau N, Salzburger W (2013). Expression and Sequence Evolution of Aromatase cyp19a1 and Other Sexual Development Genes in East African Cichlid Fishes. Mol. Biol. Evol..

[76] Bleil JD, Wassarman PM (1980). Structure and function of the zona pellucida: Identification and characterization of the proteins of the mouse oocyte’s zona pellucida. Dev. Biol..

[77] Shimizu S, Tsuji M, Dean J (1983). *In vitro* biosynthesis of three sulfated glycoproteins of murine zonae pellucidae by oocytes grown in follicle culture. J. Biol. Chem..

[78] Liang L, Soyal SM, Dean J (1997). FIGalpha, a germ cell specific transcription factor involved in the coordinate expression of the zona pellucida genes. Development.

[79] Wu G-C (2012). Testicular dmrt1 Is Involved in the Sexual Fate of the Ovotestis in the Protandrous Black Porgy. Biol. Reprod..

[80] Miyake Y, Sakai Y, Kuniyoshi H (2012). Molecular Cloning and Expression Profile of Sex-SpecificGenes, Figla and Dmrt1, in the Protogynous Hermaphroditic Fish, Halichoeres Poecilopterus. Zoolog. Sci..

[81] Benayoun, B. A., Dipietromaria, A., Bazin, C. & Veitia, R. A. FOXL2: At the Crossroads of Female Sex Determination and Ovarian Function. in *Forkhead Transcription Factors: Vital Elements in Biology and Medicine* (ed. Maiese, K.) 207–226, 10.1007/978-1-4419-1599-3_16(Springer New York, 2010). 10.1007/978-1-4419-1599-3_16 20429427

[82] Nakamoto M (2009). Gonadal sex differentiation and expression of Sox9a2, Dmrt1 and Foxl2 in Oryzias luzonensis. genesis.

[83] Wang D (2004). shou, Kobayashi, T., Zhou, L. yan & Nagahama, Y. Molecular cloning and gene expression of Foxl2 in the Nile tilapia, Oreochromis niloticus. Biochem. Biophys. Res. Commun..

[84] Nakamoto M, Matsuda M, Wang D-S, Nagahama Y, Shibata N (2006). Molecular cloning and analysis of gonadal expression of Foxl2 in the medaka, Oryzias latipes. Biochem. Biophys. Res. Commun..

[85] Liu Z (2007). Molecular cloning of doublesex and mab-3-related transcription factor 1, forkhead transcription factor gene 2 and two types of cytochrome P450 aromatase in Southern catfish and their possible roles in sex differentiation. J. Endocrinol..

[86] Guiguen Y, Fostier A, Piferrer F, Chang C-F (2010). Ovarian aromatase and estrogens: A pivotal role for gonadal sex differentiation and sex change in fish. Gen. Comp. Endocrinol..

[87] Kitano T, Takamune K, Kobayashi T, Nagahama Y, Abe SI (1999). Suppression of P450 aromatase gene expression in sex-reversed males produced by rearing genetically female larvae at a high water temperature during a period of sex differentiation in the Japanese flounder (Paralichthys olivaceus). J. Mol. Endocrinol..

[88] Uchida D, Yamashita M, Kitano T, Iguchi T (2004). An aromatase inhibitor or high water temperature induce oocyte apoptosis and depletion of P450 aromatase activity in the gonads of genetic female zebrafish during sex-reversal. Comp. Biochem. Physiol. Part A Mol. Integr. Physiol..

[89] Karube M (2007). Characterization and expression profile of the ovarian cytochrome P-450 aromatase (cyp19A1) gene during thermolabile sex determination in pejerrey, Odontesthes bonariensis. J. Exp. Zool. Part A Ecol. Genet. Physiol..

[90] Wu G-C (2010). Sex differentiation and sex change in the protandrous black porgy, Acanthopagrus schlegeli. Gen. Comp. Endocrinol..

[91] Zhou L, Gui JF (2010). Molecular mechanisms underlying sex change in hermaphroditic groupers. Fish Physiol Biochem.

[92] Baroiller JF, D’Cotta H (2016). The Reversible Sex of Gonochoristic Fish: Insights and Consequences. Sex. Dev..

[93] Shao, C. *et al*. Epigenetic modification and inheritance in sexual reversal of fish. *Genome Res*. 10.1101/gr.162172.113 (2014). 10.1101/gr.162172.113 PMC3975060 24487721

[94] Kobayashi Y, Horiguchi R, Miura S, Nakamura M (2010). Sex- and tissue-specific expression of P450 aromatase (cyp19a1a) in the yellowtail clownfish, Amphiprion clarkii. Comp. Biochem. Physiol. Part A Mol. Integr. Physiol..

[95] Piferrer F (2013). Epigenetics of sex determination and gonadogenesis. Dev Dyn.

[96] Ospina-Álvarez N, Piferrer F (2008). Temperature-Dependent Sex Determination in Fish Revisited: Prevalence, a Single Sex Ratio Response Pattern and Possible Effects of Climate Change. PLoS One.

[97] Sandra G-E, Norma M-M (2009). Sexual determination and differentiation in teleost fish. Rev. Fish Biol. Fish..

[98] Ogiwara K, Fujimori C, Rajapakse S, Takahashi T (2013). Characterization of Luteinizing Hormone and Luteinizing Hormone Receptor and Their Indispensable Role in the Ovulatory Process of the Medaka. PLoS One.

[99] Ijiri S (2008). Sexual Dimorphic Expression of Genes in Gonads During Early Differentiation of a Teleost Fish, the Nile Tilapia Oreochromis niloticus. Biol. Reprod..

[100] Baron D, Houlgatte R, Fostier A, Guiguen Y (2008). Expression profiling of candidate genes during ovary-to-testis trans-differentiation in rainbow trout masculinized by androgens. Gen. Comp. Endocrinol..

[101] Rodríguez-Marí, A. *et al*. Retinoic acid metabolic genes, meiosis and gonadal sex differentiation in zebrafish. *PLoS One* **8** (2013). 10.1371/journal.pone.0073951 PMC3769385 24040125

[102] Feng R (2015). Retinoic acid homeostasis through aldh1a2 and cyp26a1 mediates meiotic entry in Nile tilapia (Oreochromis niloticus). Sci. Rep..

[103] Yamaguchi T, Kitano T (2012). High temperature induces cyp26b1 mRNA expression and delays meiotic initiation of germ cells by increasing cortisol levels during gonadal sex differentiation in Japanese flounder. Biochem. Biophys. Res. Commun..

[104] Dong J (1996). Growth differentiation factor-9 is required during early ovarian folliculogenesis. Nature.

[105] Yoshinaga N (2004). Sexually dimorphic expression of a teleost homologue of Müllerian inhibiting substance during gonadal sex differentiation in Japanese flounder, Paralichthys olivaceus. Biochem. Biophys. Res. Commun..

[106] von Hofsten J, Olsson PE (2005). Zebrafish sex determination and differentiation: involvement of FTZ-F1 genes. Reprod Biol Endocrinol.

[107] Forconi M (2013). Characterization of Sex Determination and Sex Differentiation Genes in Latimeria. PLoS One.

[108] Pevny LH, Lovell-Badge R (1997). Sox genes find their feet. Curr. Opin. Genet. Dev..

[109] Sutton E (2011). Identification of SOX3 as an XX male sex reversal gene in mice and humans. J. Clin. Invest..

[110] Raverot G, Weiss J, Park SY, Hurley L, Jameson JL (2005). Sox3 expression in undifferentiated spermatogonia is required for the progression of spermatogenesis. Dev. Biol..

[111] Shin HS, An KW, Park MS, Jeong MH, Choi CY (2009). Quantitative mRNA expression of sox3 and DMRT1 during sex reversal and expression profiles after GnRHa administration in black porgy, Acanthopagrus schlegeli. Comp. Biochem. Physiol. Part B Biochem. Mol. Biol..

[112] Yao B, Zhou L, Wang Y, Xia W, Gui J-F (2007). Differential expression and dynamic changes of SOX3 during gametogenesis and sex reversal in protogynous hermaphroditic fish. J. Exp. Zool. Part A Ecol. Genet. Physiol..

[113] Jiang T, Hou C-C, She Z-Y, Yang W-X (2013). The SOX gene family: function and regulation in testis determination and male fertility maintenance. Mol. Biol. Rep..

[114] Yokoi H (2002). sox9 in a teleost fish, medaka (Oryzias latipes): Evidence for diversified function of Sox9 in gonad differentiation. Mol. Reprod. Dev..

[115] Chiang EFL (2001). Two Sox9 Genes on Duplicated Zebrafish Chromosomes: Expression of Similar Transcription Activators in Distinct Sites. Dev. Biol..

[116] Raymond CS (1998). Evidence for evolutionary conservation of sex-determining genes. Nature.

[117] Herpin A, Schartl M (2011). Dmrt1 genes at the crossroads: a widespread and central class of sexual development factors in fish. FEBS J..

[118] Kopp A (2012). Dmrt genes in the development and evolution of sexual dimorphism. Trends Genet..

[119] Yoshizawa A (2011). Zebrafish Dmrta2 regulates neurogenesis in the telencephalon. Genes to Cells.

[120] Lourenço R, Lopes SS, Saúde L (2011). Left-Right Function of dmrt2 Genes Is Not Conserved between Zebrafish and Mouse. PLoS One.

[121] Johnsen H, Andersen Ø (2012). Sex dimorphic expression of five dmrt genes identified in the Atlantic cod genome. The fish-specific dmrt2b diverged from dmrt2a before the fish whole-genome duplication. Gene.

[122] Marchand O (2000). DMRT1 expression during gonadal differentiation and spermatogenesis in the rainbow trout, Oncorhynchus mykiss. Biochim. Biophys. Acta - Gene Struct. Expr..

[123] Berbejillo J (2012). Expression and phylogeny of candidate genes for sex differentiation in a primitive fish species, the Siberian sturgeon, Acipenser baerii. Mol. Reprod. Dev..

[124] Fernandino JI (2008). Dimorphic Expression of dmrt1and cyp19a1 (Ovarian Aromatase) during Early Gonadal Development in Pejerrey, Odontesthes bonariensis. Sex. Dev..

[125] Picard MA-L (2015). The roles of Dmrt (Double sex/Male-abnormal-3 Related Transcription factor) genes in sex determination and differentiation mechanisms: Ubiquity and diversity across the animal kingdom. C. R. Biol..

[126] Higa M, Ogasawara K, Sakaguchi A, Nagahama Y, Nakamura M (2003). Role of steriod hormones in sex change of protogynous wrasse. Fish Physiol. Biochem..

[127] Wu G-C, Du J-L, Lee Y-H, Lee M-F, Chang C-F (2005). Current Status of Genetic and Endocrine Factors in the Sex Change of Protandrous Black Porgy, Acanthopagrus schlegeli (Teleostean). Ann. N. Y. Acad. Sci..

[128] Guidelines for the treatment of animals in behavioural research and teaching. *Anim. Behav*. **53**, 229–234 (1997). 10.1006/anbe.1999.1349 10640387

[129] EU, Directive 2010/63/EU of the European parliament and the council of 22 September 2010 on the protection of animals used for scientific purposes. Official Journal of the European Union L 276/33, Animal protection (2010).

[130] Metcalfe JD, Craig JF (2011). Ethical justification for the use and treatment of fishes in research: an update. J. Fish Biol..

[131] Diaz de Cerio O, Rojo-Bartolomé I, Bizarro C, Ortiz-Zarragoitia M, Cancio I (2012). 5S rRNA and Accompanying Proteins in Gonads: Powerful Markers to Identify Sex and Reproductive Endocrine Disruption in Fish. Environ. Sci. Technol..

[132] Shen Z-G, Yao H, Guo L, Li X-X, Wang H-P (2017). Ribosome RNA Profiling to Quantify Ovarian Development and Identify Sex in Fish. Sci. Rep..

[133] FastQC. In. http://www.bioinformatics.babraham.ac.uk/projects/fastqc.

[134] Scythe. In. https://github.com/vsbuffalo/scythe.

[135] Joshi, N. A. & Fass, J. N. sickle. In. https://github.com/najoshi/sickle.

[136] Bolger, A. M., Lohse, M. & Usadel, B. Trimmomatic: a flexible trimmer for Illumina sequence data. *Bioinformatics* **30** (2014). 10.1093/bioinformatics/btu170 PMC4103590 24695404

[137] Schmieder R, Edwards R (2011). Quality control and preprocessing of metagenomic datasets. Bioinformatics.

[138] Grabherr MG (2011). Trinity: reconstructing a full-length transcriptome without a genome from RNA-Seq data. Nat. Biotechnol..

[139] Li, B. & Dewey, C. N. RSEM: accurate transcript quantification from RNA-Seq data with or without a reference genome. *BMC Bioinformatics* **12** (2011). 10.1186/1471-2105-12-323 PMC3163565 21816040

[140] Haas BJ (2013). De novo transcript sequence reconstruction from RNA-seq using the Trinity platform for reference generation and analysis. Nat. Protoc..

[141] Li, H. *et al*. The Sequence Alignment/Map format and SAMtools. *Bioinformatics* **25** (2009). 10.1093/bioinformatics/btp352 PMC2723002 19505943

[142] Simão, F. A., Waterhouse, R. M., Ioannidis, P., Kriventseva, E. V & Zdobnov, E. M. BUSCO: assessing genome assembly and annotation completeness with single-copy orthologs. *Bioinformatics*10.1093/bioinformatics/btv351 (2015). 10.1093/bioinformatics/btv351 26059717

[143] Altschul SF, Gish W, Miller W, Myers EW, Lipman DJ (1990). Basic local alignment search tool. J. Mol. Biol..

[144] Lagnel J, Tsigenopoulos CS, Iliopoulos I (2009). NOBLAST and JAMBLAST: New Options for BLAST and a Java Application Manager for BLAST results. Bioinformatics.

[145] Hunter S (2009). InterPro: the integrative protein signature database. Nucleic Acids Res..

[146] Rice P, Longden I, Bleasby A (2016). EMBOSS: The European Molecular Biology Open Software Suite. Trends Genet..

[147] Myhre S, Tveit H, Mollestad T, Lægreid A (2006). Additional Gene Ontology structure for improved biological reasoning. Bioinformatics.

[148] GOSlim. In. http://geneontology.org/page/go-slim-and-subset-guide.

[149] Kanehisa M, Goto S (2000). KEGG: Kyoto Encyclopedia of Genes and Genomes. Nucleic Acids Res..

[150] Bourgon R, Gentleman R, Huber W (2010). Independent filtering increases detection power for high-throughput experiments. Proc. Natl. Acad. Sci. USA.

[151] Love, M. I., Huber, W. & Anders, S. Moderated estimation of fold change and dispersion for RNA-seq data with DESeq. 2. *Genome Biol* **15** (2014). 10.1186/s13059-014-0550-8 PMC4302049 25516281

[152] Gentleman, R. C. *et al*. Bioconductor: open software development for computational biology and bioinformatics. *Genome Biol* **5** (2004). 10.1186/gb-2004-5-10-r80 PMC545600 15461798

[153] Jolliffe, I. T. *Principal component analysis*. (Springer, 2002).

[154] Warnes, G. R. *et al*. gplots: Various R programming tools for plotting data. *R Packag. version* **2** (2009).

[155] Yates, A. *et al*. Ensembl 2016. *Nucleic Acids Res*. 10.1093/nar/gkv1157 (2015). 10.1093/nar/gkv1157 PMC4702834 26687719

[156] Overbeek R, Fonstein M, D’Souza M, Pusch GD, Maltsev N (1999). The use of gene clusters to infer functional coupling. Proc. Natl. Acad. Sci. USA.

