# Supplementary material for: The transcriptomic signature of different sexes in two protogynous hermaphrodites: Insights into the molecular network underlying sex phenotype in fish
Source: Sci Rep. 2018 Feb 23;8:3564. doi: 10.1038/s41598-018-21992-9 (PMC5824801; doi:10.1038/s41598-018-21992-9)
Supplement: Supplementary file 1 — Supplementary Information [file 41598_2018_21992_MOESM1_ESM.pdf]

The transcriptomic signature of different sexes in two protogynous hermaphrodites: Insights into the molecular network underlying sex phenotype in fish

Tsakogiannis, A.[1,2], Manousaki, T.[1], Lagnel, J.[1], Sterioti, A.[1], Pavlidis, M.[2], Papandroulakis, N.[1], Mylonas, C.C.[1] and Tsigenopoulos, C.S.[1*]

[1]*Institute of Marine Biology, Biotechnology and Aquaculture (IMBBC), Hellenic Centre for Marine Research (H.C.M.R.), Heraklion, Greece*

[2] *Department of Biology, University of Crete, Greece*

**Corresponding author*

Table ST1. BUSCO metrics for red porgy and common pandora transcriptome assemblies produced by Trinity

Assemblies	%Complete	%Duplicated	%Fragmented	%Missing
**Red porgy Trinity assembly**	86	33	3.8	10
**Common pandora Trinity assembly**	82	31	5.9	11

Table ST2. Red porgy and common pandora annotation summary

	Annotation Summary							
	Red porgy				Common pandora			
	Contigs	% contigs	Genes	% genes	Contigs	% contigs	Genes	% genes
	98,012		67,132		141,309		98,211	
BLAST	43,123	44	25,997	39	50,106	35	29,548	30
InterPro	38,354	39	22,691	34	44,392	31	25,876	26
With IPR number	34,858	36	20,692	31	39,792	28	23,209	24
With >=1GO	28,183	29	16,762	25	31,735	22	18,506	19
Blast2Go annotated	38,265	39	23,234	35	51,026	36	31,526	32
EC	9,572	10	5,912	9	12,821	9	7,882	8
KEGG pathways	378				377			

Supplementary Figure SF1.

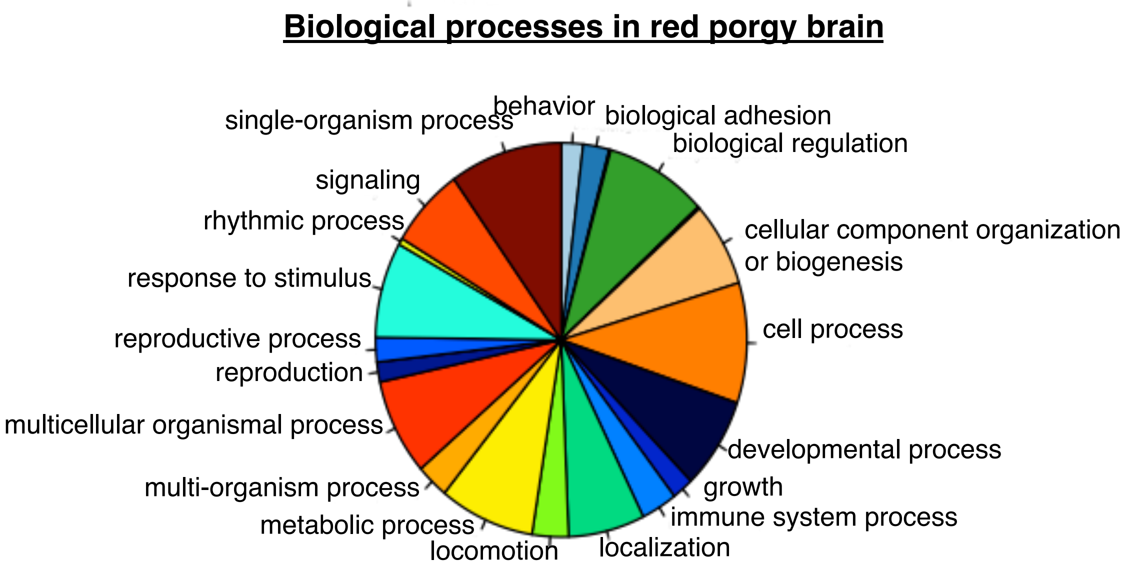

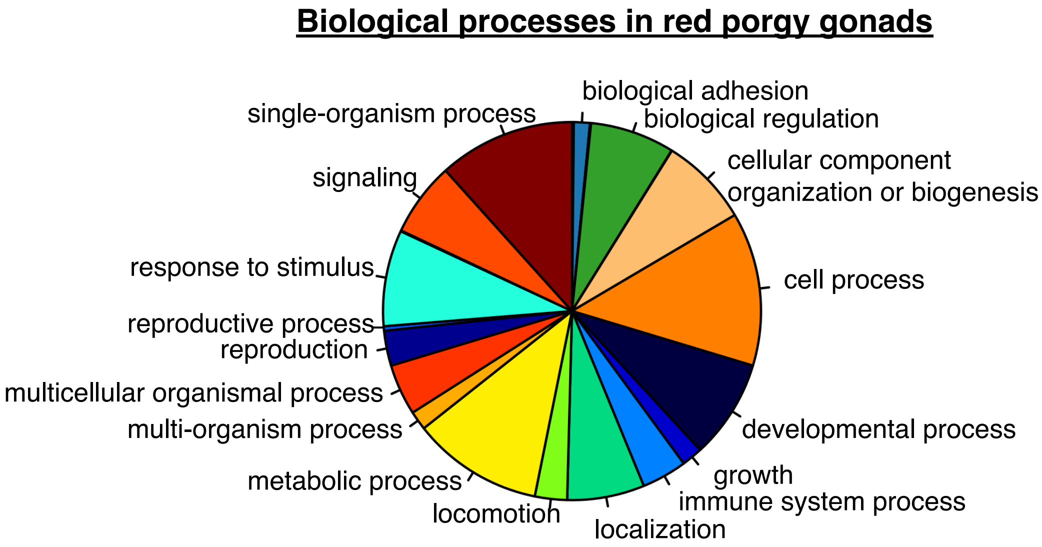

Supplementary Figure SF2.

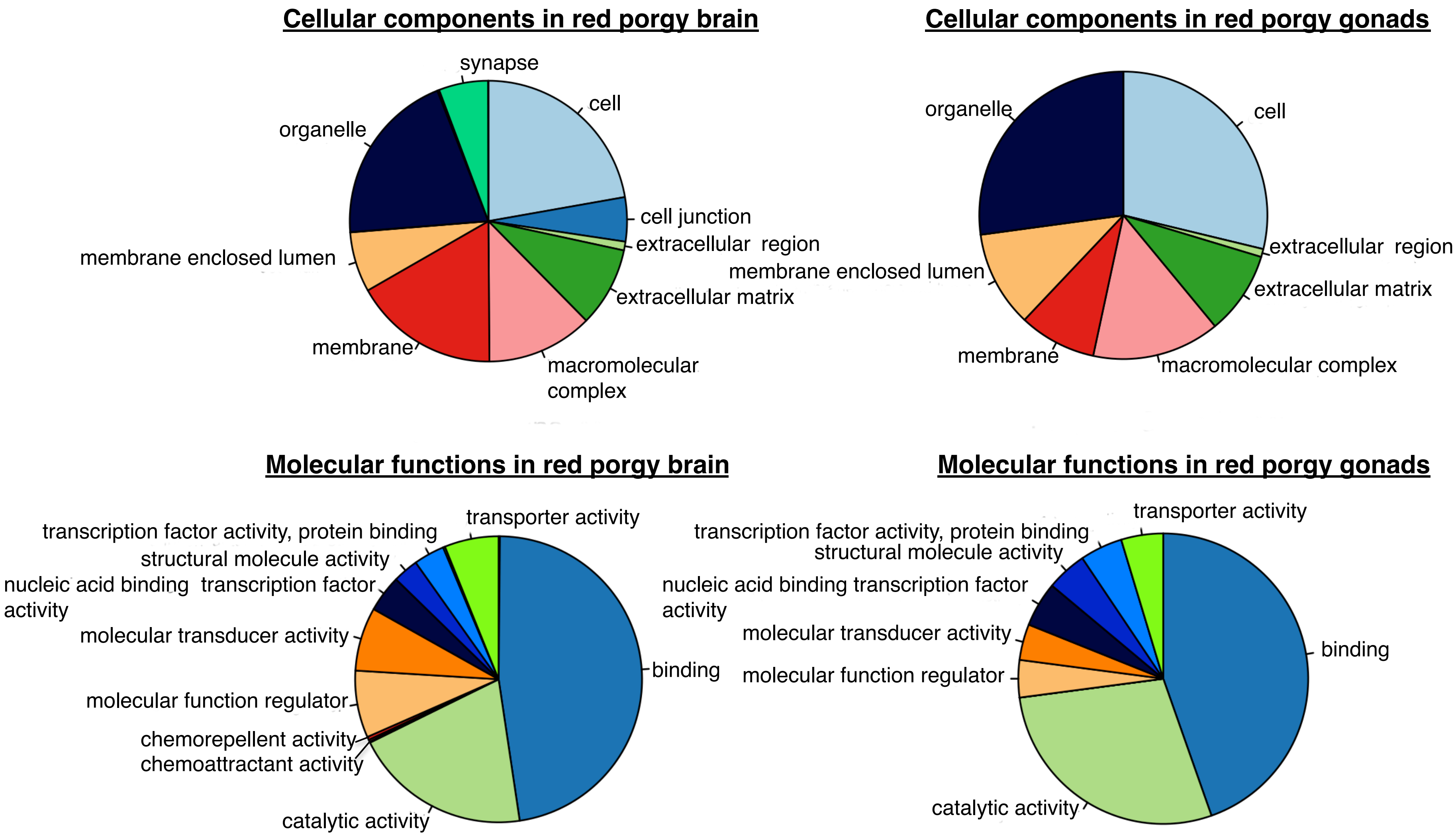

Supplementary Figure SF3.

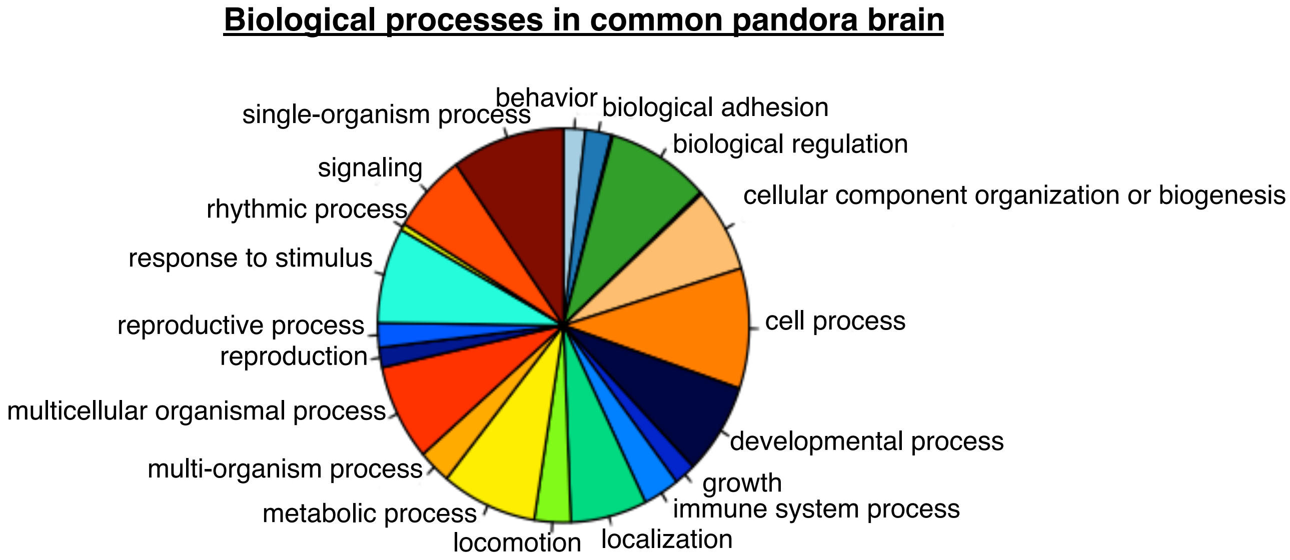

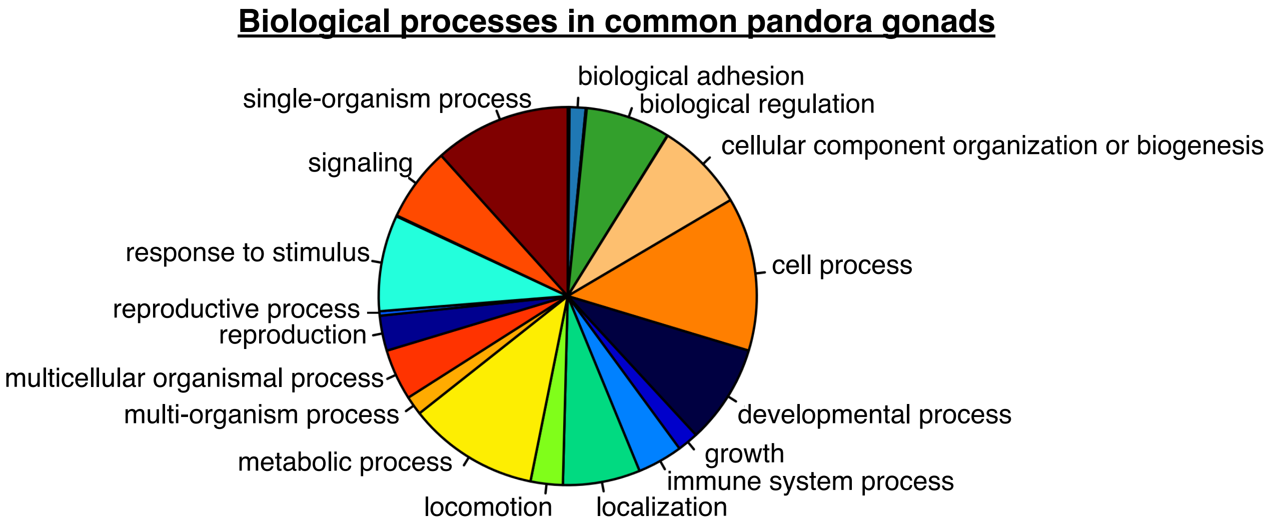

Supplementary Figure SF4.

Cellular components in common pandora brain

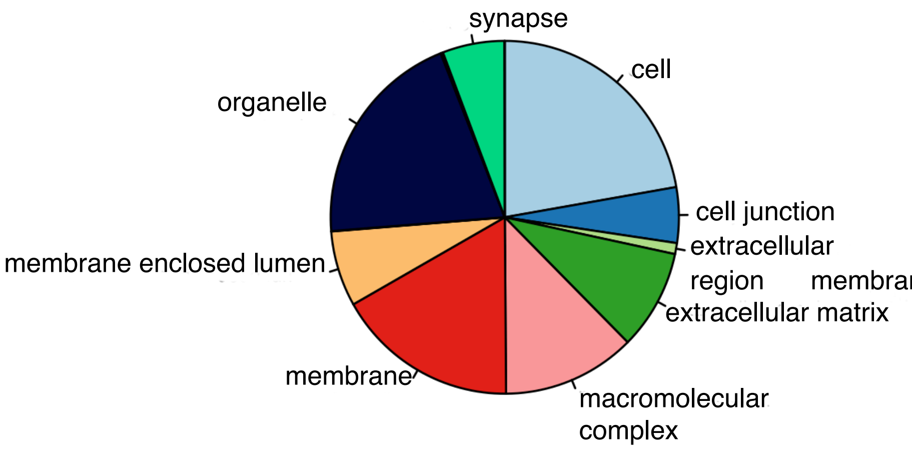

Cellular components in common pandora gonads

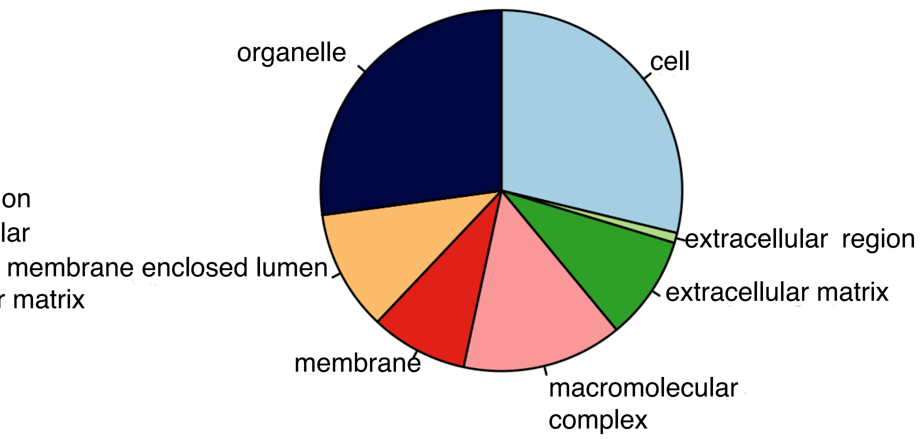

Molecular functions in common pandora brain

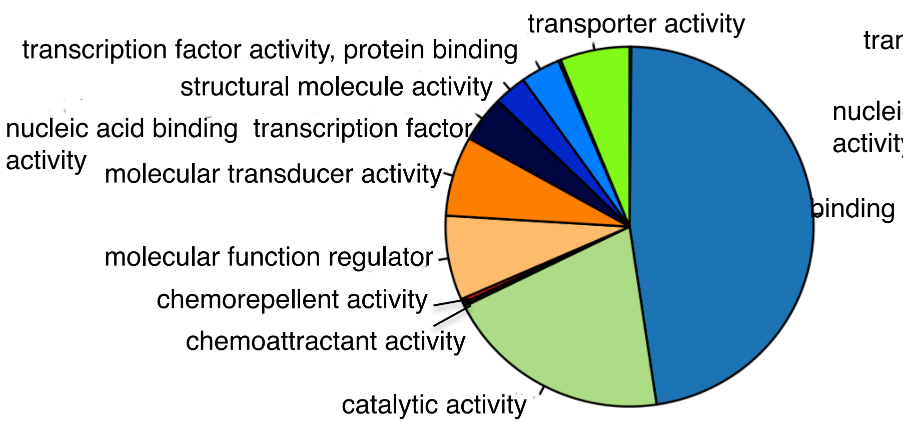

Molecular functions in common pandora gonads

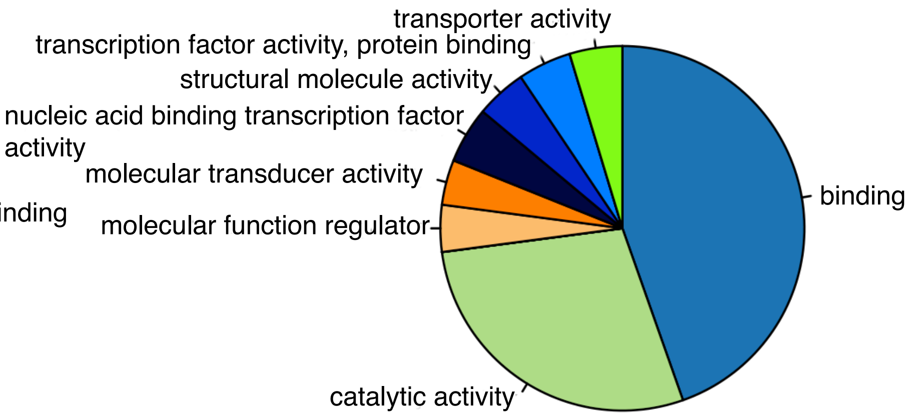

Supplementary Figure SF5.

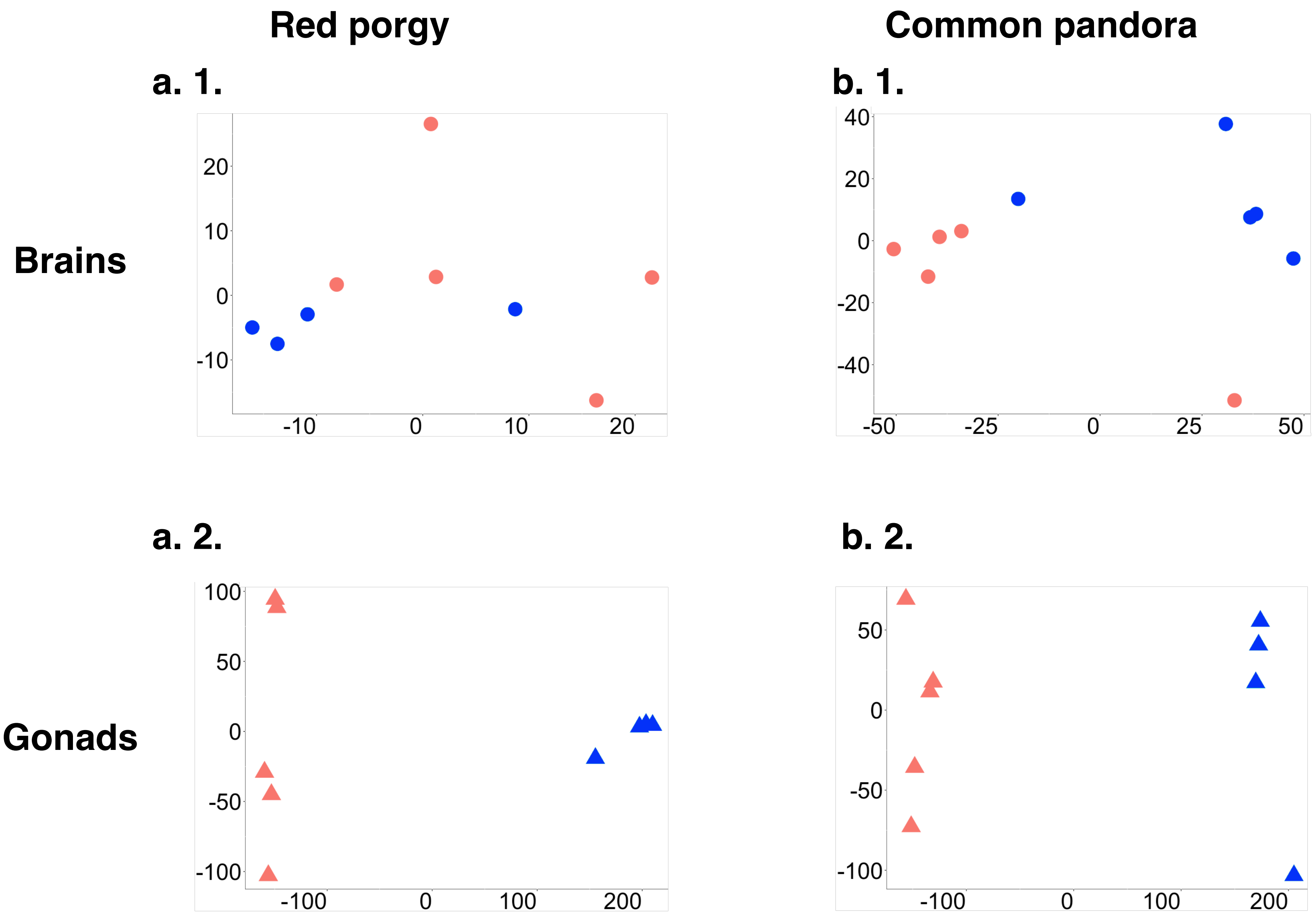

Supplementary Figure SF5. Tissue-specific principal component analysis of the DE genes in males (blue) and females (red) of two protogynous sparids. Expression profiles separately for brain (dots/circles) and gonadal (triangles) samples of red porgy and common pandora in a PCA plot. Blue color represents males and red the females. To produce each plot all reported DE genes used in each case. (a. 1.) red porgy brain samples (PC1=22% variance and PC2=17% variance), (b. 1.) common pandora brain samples (PC1=35% variance and PC2=11% variance), (a. 2.) red porgy gonad samples (PC1=68% variance and PC2=8% variance), (b. 2.) common pandora gonad samples (PC1=61% variance and PC2=7% variance).

Supplementary Figure SF6.

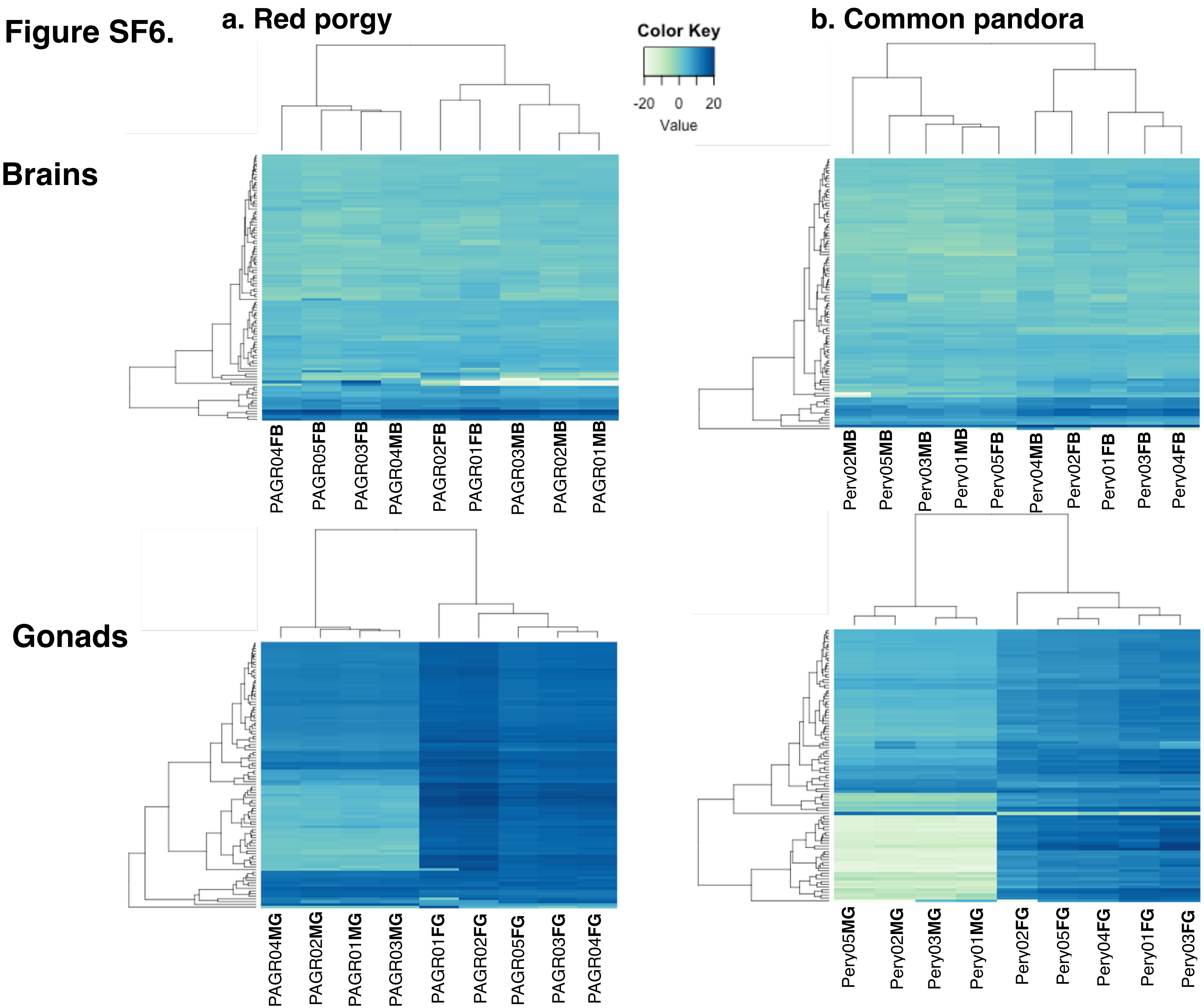

Supplementary Figure SF6. Tissue-specific heatmaps of the DE genes in (a.) Red porgy and (b.) Common pandora. Heatmaps of the variance-stabilized transformed count data of brain (on top) and gonad (bottom) samples for the differentially expressed genes between male and female individuals. Note that only the top 100 genes are included for visibility reasons.
